# Circulating Extracellular Vesicles Express Receptor Activator of Nuclear Factor κB Ligand and Other Molecules Informative of the Bone Metabolic Status of Mouse Models of Experimentally Induced Osteoporosis

**DOI:** 10.1007/s00223-022-01032-5

**Published:** 2022-10-25

**Authors:** Alfredo Cappariello, Maurizio Muraca, Anna Teti, Nadia Rucci

**Affiliations:** 1 Research Laboratories, Department of Onco-Haematology, IRCCS Bambino Gesù Children’s Hospital, Rome, Italy; 2 Department of Biotechnological and Applied Clinical Sciences, University of L’Aquila, Via Vetoio – Coppito 2, 67100 L’Aquila, Italy; 3 Department of Women’s and Children’s Health, University of Padua, Padua, Italy

**Keywords:** Extracellular vesicles, Osteoporosis, Ovariectomy, Hindlimb suspension, RANKL

## Abstract

Extracellular vesicles (EVs) are potent means of cell-to-cell communication. They are released in biological fluids, including blood, urine, and saliva, and can be exploited to identify new biomarkers of diseases. We hypothesized that EVs contain molecular cargos involved in bone metabolism, possibly mirroring biological differences between postmenopausal and disuse osteoporosis. We tested this hypothesis in primary murine osteoblasts subjected to steroid depletion or to unloading, and in the serum of animal models of osteoporosis induced by ovariectomy or hindlimb tail suspension. EVs were isolated by ultracentrifugation and analysed by transmission electron microscopy, cytofluorimetry, immunoblotting and RT-PCR. Large-scale analyses were performed by Real-Time arrays and Proteome Profiler™ Antibody arrays. Finally, precise titration of analytes was carried out by ELISA assay. In vitro*,* we confirmed an increased release of EVs enriched in surface RANKL by primary mouse osteoblasts subjected to steroid depletion or simulated microgravity compared to controls. In vivo, circulating EVs isolated from the sera of control female mice expressed RANKL along with other genes associated with bone metabolism. Serum EVs from ovariectomized or hindlimb tail-suspended mice showed distinct molecular profiles. They expressed RANKL with different kinetics, while transcriptomic and proteomic profiles uncovered unique molecular signatures that discriminated the two conditions, unveiling exclusive molecules expressed in time- and osteoporosis type-dependent manner. These results suggest that circulating EVs could represent a new tool for monitoring the onset and the progression of diverse types of the disease in mice, paving the way for their exploitation to diagnose human osteoporosis in liquid biopsies.

**Supplementary Information:**

The online version contains supplementary material available at 10.1007/s00223-022-01032-5.

## Introduction

Extracellular vesicles (EVs) recently emerged as potent means of cell–cell communication both in physiological and pathological conditions [1–5]. They are known since the late 1960s and were first discovered in the skeleton, associated with the cartilage mineralising matrix, where they were called matrix vesicles [6]. EVs are now considered essential means of information exchange in multicellular organisms [7]. They are limited by a biological membrane, cannot replicate and miss a functional nucleus [8]. There are many types of vesicles released by cells differing in size, molecular content, release pattern and mechanisms of interaction with target cells, all grouped in the term EVs [5, 8]. In osteoblasts, EVs are known to carry osteogenic and osteoclastogenic cytokines [9, 10], suggesting their involvement in the regulation of both bone formation and bone resorption. In addition, osteoblast matrix vesicles are specialized EVs enriched in alkaline phosphatase, nucleotide pyrophosphatase and pyrophosphate transporter, promoting matrix mineralization [11]. Studies on osteocyte EVs are less prominent, but there is evidence that osteocyte-like cells release EVs and that in mechanical loading, this release is Ca^2+^-dependent [12]. Typical molecular panel of osteocyte EVs is represented by sclerostin, receptor activator of NF-kB transcription factor ligand (RANKL) and osteoprotegerin (OPG) [12], suggesting their contribution in the regulation of bone remodelling. Less is known instead about osteoclast EVs. They express membrane-bound RANK, which is thought to exert a decoy role over the canonical RANK/RANKL pathway [13]. Importantly, EVs are released and identifiable also in biological fluids, including blood, urine, saliva, amniotic, spermatic, cerebrospinal and ascites fluids. Due to their biologic properties, they are considered carriers of new biomarkers of diseases to be exploited for diagnostic purposes [14].

Osteoporosis is one of the most common bone syndromes and major age-related diseases [15] characterized by low bone mass and microarchitectural deterioration of the bone tissue [15, 16]. The major types of osteoporosis in humans are primary postmenopausal osteoporosis and secondary senile osteoporosis caused by senescence, disuse and immobilization [17]. Numerous pathways are deregulated in both forms of the disease [15]. For instance, pro-inflammatory cytokines were found to play pivotal roles increasing bone resorption and/or decreasing bone formation [18–22]. Among them, RANKL is reported to be the most potent pro-osteoclastogenic protein expressed by osteoblasts, osteocytes and activated lymphocytes thus contributing to enhance the bone resorption by osteoclasts, which in turn reduces bone mass [23–25].

Osteopenia arising from different biological events, such as oestrogen depletion or disuse, is controlled by different factors and has different clinical features. Oestrogen depletion causes postmenopausal osteoporosis, in which bone loss occurs most prominently in the cancellous bone and on the endocortical surface because of increased bone turnover and negative calcium balance [26, 27]. This is in line with the role of oestrogen in the stimulation of bone formation while protecting from excess of bone resorption [28]. In disuse osteoporosis, different processes seem to be involved. In fact, immobilization induces the activation of the osteocyte network which senses the unloading and enhances bone resorption inhibiting bone formation, with mechanisms that are different and independent of those causing postmenopausal osteoporosis. Previous studies by our group demonstrated that the adipokine Lipocalin 2 was expressed by the osteogenic lineage under unloading conditions in mice and increased in the sera of unloaded mice and bed-resting human volunteers. The increase in the osteogenic lineage impaired osteoblast differentiation and enhanced the expression of interleukin (IL-6) and RANKL, which in turn stimulated bone resorption [22].

Diagnosis of postmenopausal and disuse osteoporosis would greatly benefit from specific tests to distinguish between the two forms [29–32] and from new means to easily and consistently monitor the disease and follow up the clinical management [31–33]. In this study, we propose that EVs may be exploited as a new diagnostic means that not only may allow a generic diagnosis of osteoporosis but could also discriminate the two forms described above, representing a valid tool to facilitate the monitoring of the response to specific therapies. Therefore, we investigated in a preclinical model whether EVs mirror the biological differences of postmenopausal and disuse osteoporosis and if they can be predicted to carry biomarkers to differentiate them in liquid biopsies.

## Materials and Methods

### Animals

Procedures involving animals and their care were conducted in conformity with national and international laws and policies (European Economic Community Council Directive 86/609, OJ L 358, 1, December 12, 1987; Italian Legislative Decree 4.03.2014 n.26, *Gazzetta Ufficiale della Repubblica Italiana* no. 61, March 4, 2014; National Institutes of Health guide for the Care and Use of Laboratory Animals, National Institutes of Health Publication no. 85-23, 1985) and were approved by the Institutional Review Board of the University of L’Aquila.

Twelve-week-old CD1 mice were used to set up the isolation and characterization of serum EVs. Nine-week-old CD1 mice were used to test serum EVs as osteoporosis diagnostic tools. Mice were purchased from Charles River and used according to the Animal Research Reporting of In Vivo Experiment (ARRIVE) principles (Table S1). Mice were housed in the animal facility of the University of L’Aquila, Italy, at the following conditions: temperature: 20–24 °C; humidity: 60% ± 5%; dark/light cycle: 12/12 h. They had access to food and water ad libitum and were fed a standard diet (#4RF21, Mucedola, Milan, Italy) composed of 60.8% carbohydrates, 21% proteins, 3.45% fat, 6.8% fibres, 7.95% trace elements, and 12% humidity. For these studies, mice were littermates and were randomized via online tool (True Random Number Generator, https://www.random.org/). The randomization process was conducted in double-blind. No adverse events were observed during the experiment, except for one sham-operated and two OVX mice that did not survive post-surgery.

### Rotating Wall Vessel (RWV) Bioreactor

The RWV bioreactor (model STLV, size 55 ml; Synthecon CELLON S.ar.l, Strassen, Luxembourg) is a horizontal rotating, bubble‐free culture vessel with membrane diffusion gas exchange, where the culture medium and the cells on microcarriers rotate inside the vessel with very low fluid stress forces [34–36]. Cells are attached to microcarriers (CultiSpher‐G^®^, Percell Biolytica, Åstorp, Sweden), which are maintained in suspension and rotate inside the vessel of the bioreactor as a solid body. Moreover, the horizontal rotation of the vessel subjects the cells to a constant randomization of the normal gravity vector, thus mimicking a state of simulated free fall similar to microgravity. The use of microcarrier beads is strongly suggested by the manual of the RWV bioreactor especially for cultures of adhesive cells, such as the osteoblasts.

### Primary Osteoblast Cultures

Calvariae from 7‐day‐old female CD1 mice were removed and processed by sequential collagenase/trypsin digestion as previously described [37]. The cells obtained from the second and third digestions were positive for the osteoblast marker alkaline phosphatase and expressed the osteoblast-specific genes *alkaline phosphatase (Alp), Runt-related transcription factor 2 (Runx2)* and *type I collagen a1 (CollA1)* chain. At confluence, cells were collected and resuspended in Dulbecco’s Modified Minimum Essential Medium (DMEM, Gibco, Grand Island, NY, USA) + 10% Foetal Bovine Serum (FBS, Gibco) containing CultiSpher‐G^®^ microcarriers (1 g/l medium), at a cellular density of 1.2 × 10^6^ cells/ml, to allow cell–microcarrier interaction. This suspension was grown for 5 days into the RWV bioreactor under 16‐rpm rotation, which leads to a simulated microgravity condition of 0.008* g*, or in non‐adhesive Petri dishes at unit gravity condition (1* g*), in DMEM plus FBS, or charcoal-stripped FBS (Gibco) to reduce steroids, including oestrogen (mean oestrogen concentration: unstripped FBS 28.25 ± 9.6 pg/ml; stripped FBS 8.082 ± 5.12 pg/ml). After 5 days, cells were starved in FBS-free medium to avoid the contamination of culture media with EVs present in FBS [5] and incubated for further 24 h in the same conditions. At the end of the experiments, media were harvested and subjected to EV isolation, while cells were collected for RNA or protein extraction.

### In Vivo* Models of Osteoporosis*

Ten ovariectomized (OVX) and ten sham-operated 9-week-old female CD1 mice were housed under normal cage conditions and euthanized by CO_2_ inhalation after 10 weeks from OVX. Uteri were removed from sham-operated and OVX mice to evaluate their weights. One sham-operated and two OVX mice did not survive the operation and were not included in the study.

Hindlimb tail suspension (HL-TS) was performed in ten 9‐week‐old CD1 female mice according to the protocol described by Sakata et al. [38]. Briefly, a strip of elastic tape forming a half‐circle at the middle of the tail was applied to the ventral surface of the mice tail. A swivel attached to the half‐circle of the tape was fixed to an overhead wire, the height was adjusted to maintain the mice suspended at an approximately 30° angle. The swivel apparatus allowed animals to move freely into the cage using their forelimbs. Ten female CD1 mice were maintained under normal cage conditions as controls. All animals were euthanized by CO_2_ inhalation after 10 weeks from the start of HL-TS.

Hindlimbs were removed from all groups of mice, cleaned from soft tissues, fixed in 10% buffered formalin, and then passed into 70% ethanol for microtomography (µCT) evaluation.

### EVs Isolation

EVs were obtained according to [5, 8, 37, 39]. To isolate EVs from osteoblasts, upon reaching 80% confluence, osteoblast cultures were washed in PBS and starved in FBS-free DMEM. After 24 h, culture media were harvested and sequentially centrifuged at 300* g* and 4 °C for 5 min to remove dead cells, and at 3000* g* and 4 °C for 15 min to eliminate debris. The supernatants were then transferred to a Beckman L7-65 ultracentrifuge (Beckman Coulter, Indianapolis, IN, USA) in a Beckman SW41-Ti rotor and ultracentrifuged at 8000* g* and 4 °C for 20 min to remove large vesicles and apoptotic bodies. The cleared supernatants were centrifugated at 100,000* g* and 9 °C for 70 min and the resulting pellets, containing EVs, were resuspended in PBS or in extraction buffers according to the experimental protocol.

To isolate circulating EVs, the blood was harvested thrice from control, OVX and HL-TS living mice through the submandibular vein close to the cheek pouches, alternating cheek sides, at 5, 7, and 10 weeks from the induction of osteoporosis. The blood was centrifugated at 1500* g* and 4 °C for 15 min. Sera were then recovered and sequentially centrifuged and ultracentrifuged as described above, then the pellets containing EVs were resuspended in PBS or in extraction buffers according to the experimental protocol.

### Transmission Electron Microscopy (TEM)

For TEM analysis, 5 μL of EV suspension in PBS was deposited onto formvar-coated grids and left to air dry for 20 min. Grids were then washed with PBS and fixed in 1% glutaraldehyde for 5 min. After washing with distilled water, samples were contrasted with uranyl-oxalate solution (4% uranyl acetate, 0.15 M oxalic acid, pH 7, Sigma–Aldrich, St. Louis, MO, USA) for 5 min. Finally, grids were air dried for 10 min and observed under a Philips CM 30 TEM at 80 kV.

### Flow Cytometry

The EV pellets from both primary osteoblasts and sera were incubated for 30 min at 37 °C with the membrane-permeant green dye 5-ChloroMethyFfluoresceinDiAcetate (CMFDA, #C7025, Thermo Fisher Scientific, Waltham, MA, USA) then incubated with a PhycoErythrin (PE)-conjugated anti-RANKL antibody (1:100; #560295, BD Biosciences; Erembodegem, Belgium) for 30 min at 4 °C. The immunophenotype of EVs was evaluated by a BD FACSMelody (BD Biosciences) equipped with FACSChorus software. Post-acquisition analysis of FACS data was performed on FACSDiva software. Nanofluorescent standard particles (# NFPPS-52-4&nbsp;K, Spherotech, Lake Forest, IL, USA) were used to set dimensional gate up to 1 μm.

### Gene Expression

After removal of culture medium to retrieve EVs as described in the “EV isolation” section, donor osteoblasts were extensively washed in PBS and trypsinized to remove remnant EVs. Osteoblast suspensions were then washed twice in PBS by centrifugation at 300* g* and 4 °C for 5 min, then 1 million osteoblasts and their EV pellets were processed for RNA extraction by RNeasy mini kit (#74104, Qiagen, Valencia, CA, USA), according to the manufacturer's instructions, and quantified using a NanoDrop 2000 spectrophotometer (Thermo Fisher Scientific).

For RT‐PCR, 1 µg of total RNA extracted from osteoblasts was reverse transcribed in cDNA using M‐MLV reverse transcriptase and the equivalent of 0.1 µg was employed for conventional PCR. Results, shown by electrophoresis of PCR products in a 2% agarose gel plus Gel Red staining dye, were normalized versus the housekeeping gene *Gapdh*. Primer pairs are shown in Table S2. PCR conditions were 94 °C for 30 s, 60 °C for 30 s, and 72 °C for 30 s, replicated for 27 cycles.

For large‐scale analysis, 16 ng of total RNA extracted from circulating EVs, collected from 3 samples per group selected by randomization and quantified as described above, was retrotranscribed by PreAMP cDNA Synthesis Primer Mix, then the cDNAs were ran on mouse Osteoporosis PCR arrays (#PAMM‐170Z, RT^2^ Profiler Real-Time PCR Array, Qiagen). Array data were automatically analysed by the dedicated software, RT^2^ Profiler PCR Array data analysis template v3.2 (Qiagen). Any experimental sample was analysed for quality control criteria: PCR Array Reproducibility (PPC C_T_ is 20 ± 2), Reverse Transcription Control (RTC, Delta C_T_ {AVG RTC—AVG PPC} <  = 7, Genomic DNA Contamination (GDC, C_T_ >  = 30). Cut-off for lower limit of Ct detection was set to 40. Each array was normalized by *Gapdh*, which was the most stable and reliable gene in all samples, with differences in Ct values less than 1 across all of them.

### Western Blot Analysis

Serum EVs were lysed in RadioImmunoPrecipitation Assay (RIPA) buffer (50 mM Tris–HCl, pH 8.0, with 150 mM sodium chloride, 1.0% NP-40, 0.5% sodium deoxycholate, and 0.1% sodium dodecyl sulphate, Sigma-Aldrich) and proteinase inhibitor cocktail (#P2714, Sigma-Aldrich). Protein content was quantified by the bicinchoninic acid assay (#23225, Thermo Fisher Scientific). Forty-five μg of proteins was separated by SDS-PAGE in reducing conditions, and blotted onto nitrocellulose membrane, which was incubated overnight at 4 °C with primary CD63 (1:200; #143902, BioLegend, San Diego, CA, USA), Annexin II (1:150, #sc-28385, C-10 clone, Santa Cruz Biotechnology) and RANKL (1:200, #AF462, R&D Systems, Minneapolis, MN, USA) antibodies. After 1-h incubation at room temperature with HorseRadish Peroxidase (HRP)-goat anti-rat IgG (1:2000 #sc-2006, Santa Cruz Biotechnology), bands were visualized by Super Signal West Pico chemiluminescent substrate (#RA227125, Thermo Fisher Scientific).

### Cytokine Protein Profile Analysis

Protein lysates were obtained from circulating EVs after lysis with RNeasy Kit RW buffer, according to the manufacturer protocol (Qiagen). Briefly, 130 μl of serum of each mouse was ultracentrifuged and washed in PBS twice to isolate EVs and remove the contaminants. The final EV pellet was resuspended in 20 μl RW buffer and passed through a RNeasy mini kit spin column. The first eluate of RW buffer, enriched in proteins, was subjected to protein isolation with ice-cold acetone and centrifugated at 14,000* g* and 4 °C for 10 min. The pellets were washed 4 times in PBS and centrifugated at 14,000* g* and 4 °C for 10 min. Due to the low yield of EVs for protein analysis, the last pellets of 3 samples for each experimental groups selected by randomization were pooled and added to the nitrocellulose membranes of the Mouse XL Cytokine Array Kit (#ARY028, R&D Systems), which included 111 capture antibodies printed in duplicate. The membranes were incubated overnight at 4 °C, washed three times and incubated with the detection antibody cocktail for 1 h at room temperature. After 3 washes, a 30-min incubation with the Streptavidin-HRP solution was performed, then membranes were washed again and incubated with the ChemiReagent mix for the detection of the positive spots by chemiluminescence after 1 h of exposition. For data analysis, the intensity of each spot was determined by densitometry, and the average background of internal negative control was subtracted, while the normalization of the protein expression was performed by calculating the ratio with the signal intensity of the internal positive controls.

### Enzyme-Linked Immunosorbent Assay (ELISA)

ELISA was performed on proteins isolated from circulating EVs obtained from 130 μl of serum and extracted with RIPA buffer and proteinase inhibitor cocktail (Sigma-Aldrich) or in whole sera to quantify the levels of P-selectin (#EM0160, Fine Test, Wuhan, China) or Lipocalin 2 (#MLCN20, R&D Systems). Protocols were carried out according to the instructions provided in the kits.

### Statistics

Data are reported as mean ± standard deviation (SD). Student’s *t* test was applied for comparison between two groups (*p*-value < 0.05). One-way analysis of variance (ANOVA) using non-parametric Dunnet post hoc test was used as comparative statistical method among multiple groups (*p*-value < 0.05) (GraphPad Prism 7.00; GraphPad Software, Inc., La Jolla, CA, USA). All experiments were performed with three technical replicates for each point and repeated at least three times (in vitro) or using samples from three to ten mice/group (ex vivo and in vivo). For the cytokine array assays, due to the low yield of serum EVs for protein analysis, the evaluations were performed pooling the EVs isolated from the sera of three mice per group; therefore, no statistics is available for this dataset. However, relevant results were then statistically confirmed by ELISA.

## Results

### Osteoblast EVs

**Fig. 1:**
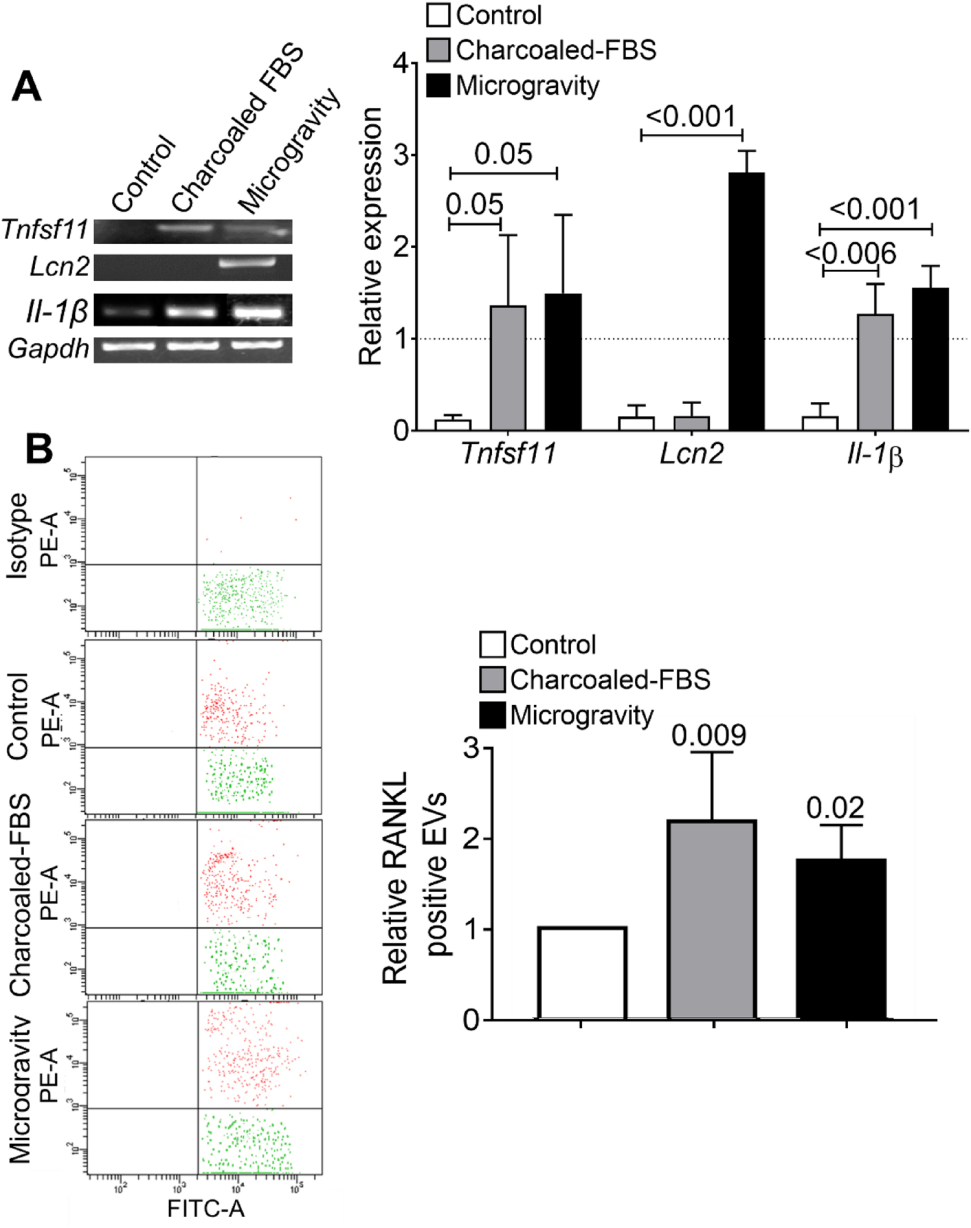
Gene and RANKL expression in primary murine osteoblast EVs. **A** Left panel: representative RT-PCR for the indicated genes performed using cDNAs from osteoblasts cultured in DMEM (Control), in DMEM + Charcoaled FBS and in microgravity condition (0.008* g*). Right panel: densitometric quantification of the genes presented in the left panel. **B** EVs isolated from osteoblasts cultured as described in panel A were loaded with CMFDA (Fluorescein isothiocyanate-antibody—FITC-A) and analysed for RANKL (Phycoerythrin-antibody—PE-A) expression. Left panels: representative dot plots of FACS-analysed EV pellets isolated from conditioned media of the osteoblast cultures evaluated in panel A. Right panel: quantification of fold changes of percentage of RANKL-positive EVs shown in the left panels. Results are the mean ± SD of independent experiments (control, microgravity: *n* = 4; charcoaled FBS: *n* = 3). Statistics: one-way ANOVA with post hoc Dunnet test for multiple comparison. *p* values on top of bars

To investigate whether the molecular profile of the EVs released by primary mouse calvarial osteoblasts was affected by in vitro conditions mimicking the cellular alterations induced by postmenopausal and disuse osteoporosis, we cultured the cells in steroid-depleted medium or under microgravity [22]. Osteoblasts, cultured for 7 days in DMEM supplemented with charcoal-stripped FBS to reduce steroids, showed an increase of *Tnfsf11* (encoding RANKL), and *Il-1β* transcriptional expression, whereas *Lcn2* was not affected (Fig. 1a). In contrast, in agreement with our previous results [15], osteoblasts cultured under microgravity for 7 days showed an increased expression of *Tnfsf11*, *Lcn2,* and *Il-1β* mRNAs (Fig. 1a). These results suggest that osteoblasts have a differential molecular response to steroid depletion and unloading.

Given that RANKL is a surface-bound protein, we established if the two conditions affected the membrane RANKL-positive EV ratio versus control cultures. Flow cytometry showed that osteoblasts incubated in DMEM supplemented with charcoal-stripped FBS or subjected to microgravity released more RANKL-positive EVs than osteoblasts cultured in control conditions (Fig. 1b). These data confirmed that EVs mirror the exacerbated pro-osteoclastogenic status of osteoblasts induced by both steroid depletion and unloading.

### Serum EV Molecular Profiles in Mice

**Fig. 2:**
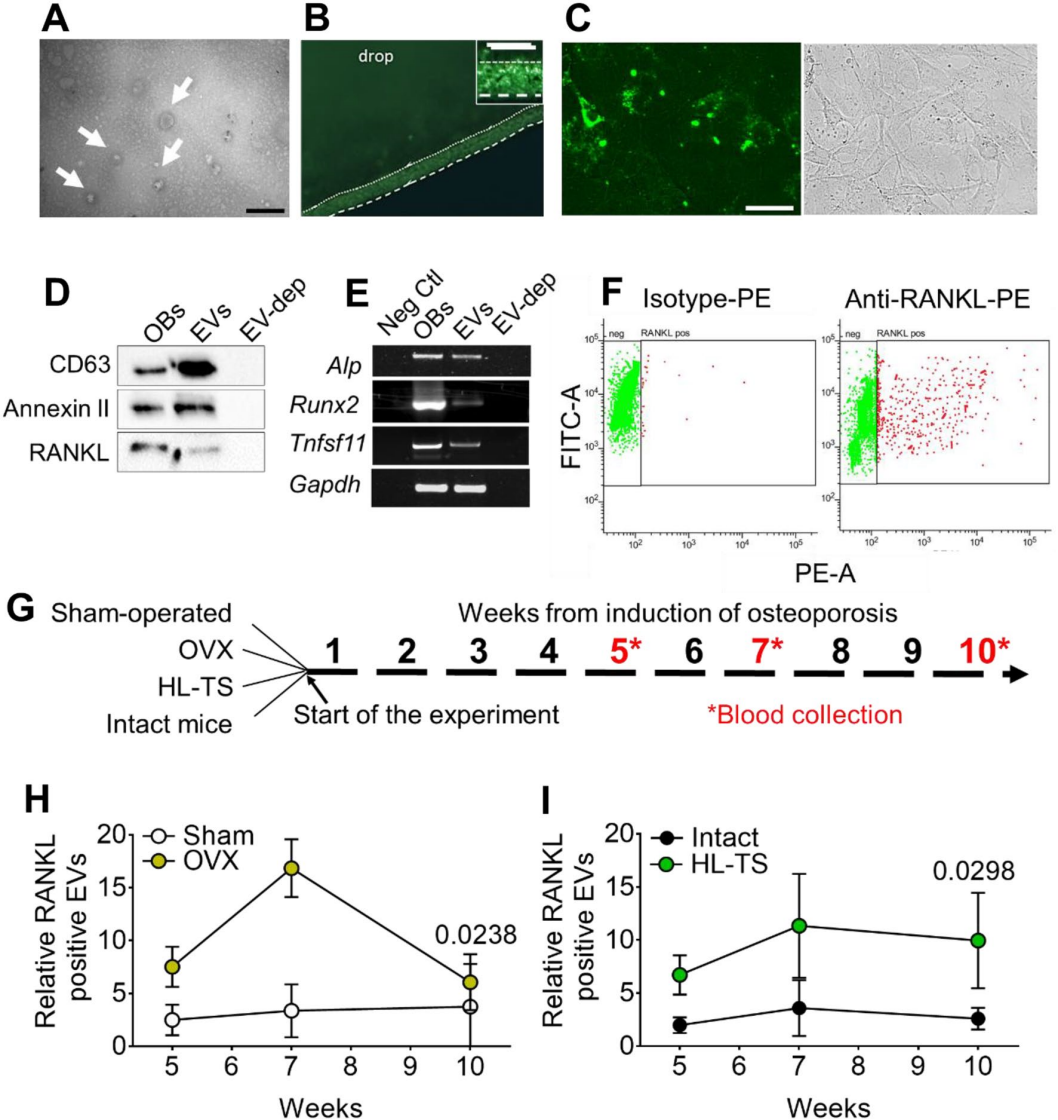
Characterization of circulating EVs. **A** Morphology of circulating EVs (arrows) observed by TEM. Scale bar = 200 nm. **B** Fluorescence microscopy of a drop of circulating EVs, isolated from sera of 1-month-old female mice, loaded with CMFDA. Dotted line: edge of meniscus of the drop. Scale bar = 30 μm. Inset: higher magnification of the drop meniscus enriched in CMFDA-positive EVs. Scale bar = 5 μm. **C** Primary murine osteoblasts were incubated for 48 h with circulating EVs loaded with CMFDA (green). Cells were observed by fluorescence microscopy for their uptake of the fluorescent CMFDA dye previously loaded into the EVs (left panel). Right panel: phase contrast image of the left panel microscopic field. Scale bar = 30 μm. **D** Western blot of the indicated proteins extracted from circulating EVs of 12-week-old female mice. OBs = protein lysates of primary murine osteoblasts; EVs = protein lysates of circulating EVs; EV-dep = ultracentrifuged supernatant depleted of EVs. **E** RT-PCR for the indicated genes in circulating EVs of 12-week-old female mice. Neg Ctl = negative control, OBs = total RNA form primary murine osteoblasts; EVs = total RNA from circulating EVs; EV-dep = total RNA from ultracentrifuged supernatant depleted of EVs. **F** Dot plot of circulating EV pellets isolated from the serum of 12-week-old female mice, loaded with CMFDA (FITC-A, green) and stained with irrelevant PE-conjugated isotype antibody (left) and PE-conjugated anti-RANKL antibody (right), analysed by FACS. **G** Cartoon illustrating the experimental procedures for induction of osteoporotic phenotype and the isolation of circulating EVs. **H** Quantification of RANKL-positive circulating EVs identified by flow cytometry at the indicated weeks from the start of the experiment in OVX and **I** HL-TS mice compared to matched controls. Results are representative (**A**–**F**) or the mean ± SD (**H**, **I**) of at least 5 animals per group, selected by randomization. Statistics: one-way ANOVA using non-parametric Dunnet post hoc test among multiple groups (whole curves OVX vs. Sham and HL-TS vs. Intact mice). *p* values are reported in the graphs

Our in vitro results prompted us to hypothesize that the molecular profile of circulating EVs could also reflect the status of bone cell activities in postmenopausal and disuse osteoporosis, respectively, thus opening an avenue to the exploitation of this circumstance as EV liquid biopsy diagnostics. To confirm this hypothesis, we first isolated and characterized EVs from the sera of 12-week-old wildtype CD1 female mice housed in standard conditions. Isolated serum EVs were observed by TEM and confirmed to exhibit the expected EV size, morphological features, and intact membranes (Fig. 2a) [40]. Serum EVs were then loaded with the permeant dye 5-chloromethylfluorescein diacetate (CMFDA), which is enzymatically de-esterified and retained by intact EVs as an impermeant probe. Drops of CMFDA-positive fluorescent EVs were observed by epifluorescence microscopy and confirmed to be intact and enzymatically active (Fig. 2b). Serum EVs were then isolated, loaded with the permeant dye CMFDA and incubated with unstained osteoblasts. Results showed that the serum EV fluorescent dye was transferred to primary mouse osteoblasts (Fig. 2c), suggesting integration of their content with the host cells. Western blot analysis of EV extracts confirmed the presence of protein cargo typical of EVs, including CD63 and Annexin II [5] as well as the expression of RANKL (Fig. 2d). Serum EVs were also evaluated by RT-PCR and found to express the osteoblast genes *Alp, Runx2* and *Tnfsf11* (Fig. 2e). Finally, flow cytometry unveiled that about 14% of serum EVs were RANKL-positive (Fig. 2f). Altogether, these results suggest that a detectable portion of circulating EVs share a molecular profile with the osteogenic lineage.

### RANKL Expression in EVs from Osteoporotic Mouse Sera

Nine-week-old CD1 female mice were subjected to OVX or HL-TS and to serum collection after 5, 7 and 10 weeks from the start of the experiments (Fig. 2g). At the end of the experiments, mice were sacrificed, and the status of oestrogen depletion was assessed in OVX mice measuring the uterus weight, which was significantly reduced by the removal of the ovaries (Fig. S1A). Furthermore, the distal femurs of all groups were subjected to µCT and morphometric measurements of bone volume over total volume that ascertained the severe osteoporotic status (Fig. S1B–E). In the two groups of mice, flow cytometry demonstrated that both conditions induced a time-dependent increase of the rate of RANKL-positive EVs in the sera, with a bell-like kinetics in OVX mice, reaching the peak at 7 weeks from OVX and declining thereafter to control values (Fig. 2h), and a sustained increase in HL-TS mice reaching the plateau at 7 weeks from the start of the tail suspension and remaining similarly high up to 10 weeks (Fig. 2i). These results suggest that circulating RANKL-positive EVs increase in both models of osteoporosis but in a manner that kinetically discriminates the outcome of oestrogen depletion from unloading.

### Messenger RNA Profile of Circulating EVs

**Fig. 3:**
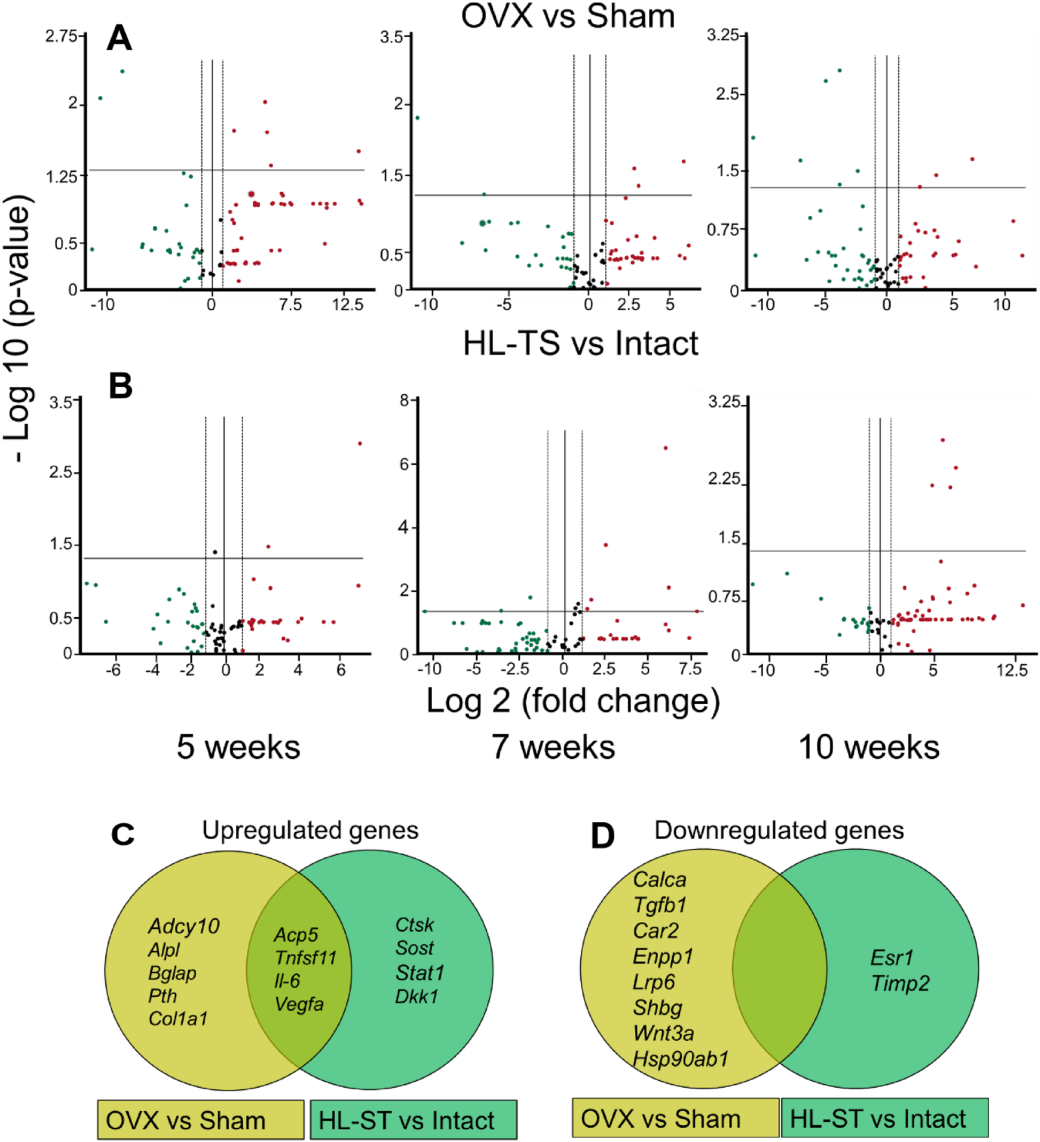
Transcriptomic analysis of circulating EVs from OVX and HL-TS mice. Volcano plots reporting up(red, right)- and down(green, left)-regulated genes in circulating EVs of **A** OVX versus Sham-operated mice and **B** HL-TS versus Intact mice at the indicated weeks from operation and tail suspension, respectively. Scatter plots represent individual mRNAs. Horizontal line = statistical limit (*p* = 0.05) of Student’s *t* test. Vertical line = fold change (FC) limit (± 2). *n* = 3 animals for each group at each time. Analysis has been run on RT^2^ Profiler PCR Array Data Analysis Template Version 3.2. **C** Venn diagrams showing the significantly up-regulated and **D** down-regulated transcripts in circulating EVs from OVX (vs. Sham) and HL-TS (vs. Intact) mice. Analysis was run on total RNAs extracted from 3 animals for each timepoint and groups

To better characterize the molecular content, we investigated the mRNA profile of serum EVs in intact, sham-operated, OVX and HL-TS mice, using an RT-PCR array with 84 genes highly related to bone metabolism. Table S3 shows the list of the detectable mRNA species in the 4 groups of mice at each timepoint investigated. Some genes were consistently expressed in all groups at the same timepoints, including *Col1a2*, *Vegfa* and the housekeeping genes *Actb* and *Gapdh.* Most genes instead showed a different pattern of expression between the groups and in the same group at different timepoints. Finally, the expression of various genes appeared inconsistent among the groups and the timepoints (Table S3). Based on these findings, we constructed volcano plots to compare the statistically significant changes of gene expression between the groups (Fig. 3a, b). Table 1 shows the genes differentially up- and down-regulated in OVX versus sham-operated mice and in HL-TS versus intact mice, whereas Table 2 shows the proteins encoded by these genes and their role in bone metabolism. Results suggest that the two conditions affected the release in the bloodstream of EVs with a limited number of differentially expressed genes (Fig. 3c, d), and that in each condition, there was a specific kinetics (Figs. S2, S3).

**Table 1:** Quantification of mRNA fold changes in circulating EVs from OVX and HL-TS mice versus control mice using the mouse Osteoporosis PCR array (#PAMM‐170Z, RT^2^ Profiler Real-Time PCR Array, Qiagen)

Gene	Fold changes	*p* value	Gene	Fold changes	*p* value	Gene	Fold changes	*p* value
OVX versus Sham-operated mice								
5^a^			7^a^			10^a^		
*Up-regulated genes*								
*Acp5*	47.61	0.044	*Acp5*	59.71	0.017	*Col1a1*	18.55	0.035
*Adcy10*	14,664.09	0.031	*Pth*	8.36	0.037	*Il-6*	155.42	0.021
*Alpl*	4.21	0.019	*Tnfsf11*	7.01	0.021	*Vegfa*	7.09	0.049
*Bglap*	31.85	0.009						
*Tnfsf11*	36.67	0.019						
*Down-regulated genes*								
*Tgfb1*	− 1520.15	0.008	*Car2*	− 1820.35	0.004	*Car2*	− 2574.36	0.011
*Calca*	− 362.04	0.004	*Enpp1*	− 99.73	0.049	*Lrp6*	− 15.89	0.046
						*Shbg*	− 36.17	0.002
						*Wnt3a*	− 15.89	0.002
						*Hsp90ab1*	− 160.9	0.022

HL-TS versus intact mice								
5^a^			7^a^			10^a^		
*Up-regulated genes*								
*Acp5*	174.05	0.001	*Acp5*	2.89	0.022	*Acp5*	28.44	0.007
*Tnfsf11*	5.44	0.034	*Ctsk*	2.47	0.042	*Dkk1*	92.2	0.008
			*Il-6*	58.76	3.5 × 10^–7^	*Il-6*	130.69	0.004
			*Sost*	66.56	0.01	*Vegfa*	56.36	0.002
			*Stat1*	209.38	0.05			
			*Vegfa*	5.23	4.1 × 10^–4^			
*Down-regulated genes*								
			*Esr1*	− 13.12	0.05			
			*Timp2*	− 4.06	0.02			

^a^Weeks from the start of the experiment

**Table 2:** Proteins encoded by the genes presented in Table 1, function and role in bone metabolism

Gene	Protein	Function	Role in bone metabolism
*Acp5*	Tartrate resistant acid phosphatase type 5	Phosphate ester hydrolytic enzyme	Substrate dephosphorylation during osteoclast bone resorption
*Adcy10*	Adenylyl cyclase 10	Soluble intracellular enzyme sensing bicarbonate levels	Osteoclast intracellular pH regulation
*Alpl*	Alkaline phosphatase	Dephosphorylating enzyme	Osteoblast matrix mineralization
*Bglap*	Osteocalcin	Bone matrix gla-protein	Osteoblast matrix mineralization and regulation of energy metabolism
*Calca*	Calcitonin/Calcitonin-Related Polypeptide, Alpha	Encodes calcitonin, calcitonin gene-related peptide and katacalcin	Calcitonin, osteoclast inhibiting hormone
*Col1a1*	Collagen type 1 a1 chain	Subunit of type 1 tropocollagen	Constituent of 95% of the bone organic matrix
*Ctsk*	Cathepsin K	Acidic type I collagenolytic enzyme	Collagen type I degradation during osteoclast bone resorption
*Dkk1*	Dickkopf-related protein 1,	Wnt pathway inhibitor	Inhibitor of osteoblast activity
*Enpp1*	Ecto-nucleotide pyrophosphatase/phosphodiesterase	Transmembrane dephosporylating enzyme	Insulin receptor signalling and bone matrix mineralization
*Il-6*	Interleukin 6,	Pro-inflammatory cytokine	Pro-osteoclastogenic and anti-osteoblastogenic
*Lrp6*	low-density lipoprotein receptor-related protein 6	Key component of the Wnt pathway	Osteoblast activity enhancer
*Ltbp2*	Latent-transforming growth factor beta-binding protein 2	TGFβ sequestering protein	TGF β activity blocker
*Pth*	Parathyroid hormone	Calcium/phosphate regulating hormone	Inducer of RANKL expression in osteoblasts favouring osteoclastogenesis
*Sost*	Sclerostin	Antagonist of the LRP5/LRP6 receptor	Osteoblast activity inhibitor
*Tgfb1*	Transforming Growth Factor β 1	Pleiotropic cytokine	Coupling of bone resorption with bone formation
*Timp2*	Tissue inhibitor of metalloproteinases 2	Matrix metalloproteinase inhibitor	Regulator of bone matrix degradation
*Tnfsf11*	RANKL	Osteoclastogenic cytokine	Inducer of osteoclastogenesis
*Vegfa*	Vascular endothelial growth factor	Angiogenic cytokine	Stimulator of angiogenesis and osteoclastogenesis
*Wnt3a*	Member of Wnt family	Secreted signalling protein	Enhancer of osteoblast activity through β-catenin signalling

### Protein Profile of Circulating EVs

We next evaluated the cytokine/growth factors cargo of serum EVs in the two osteoporotic models. The profile overview is reported in Fig. S4 while the list of identified proteins and their quantification at each time point is reported in Table S4.

**Fig. 4:**
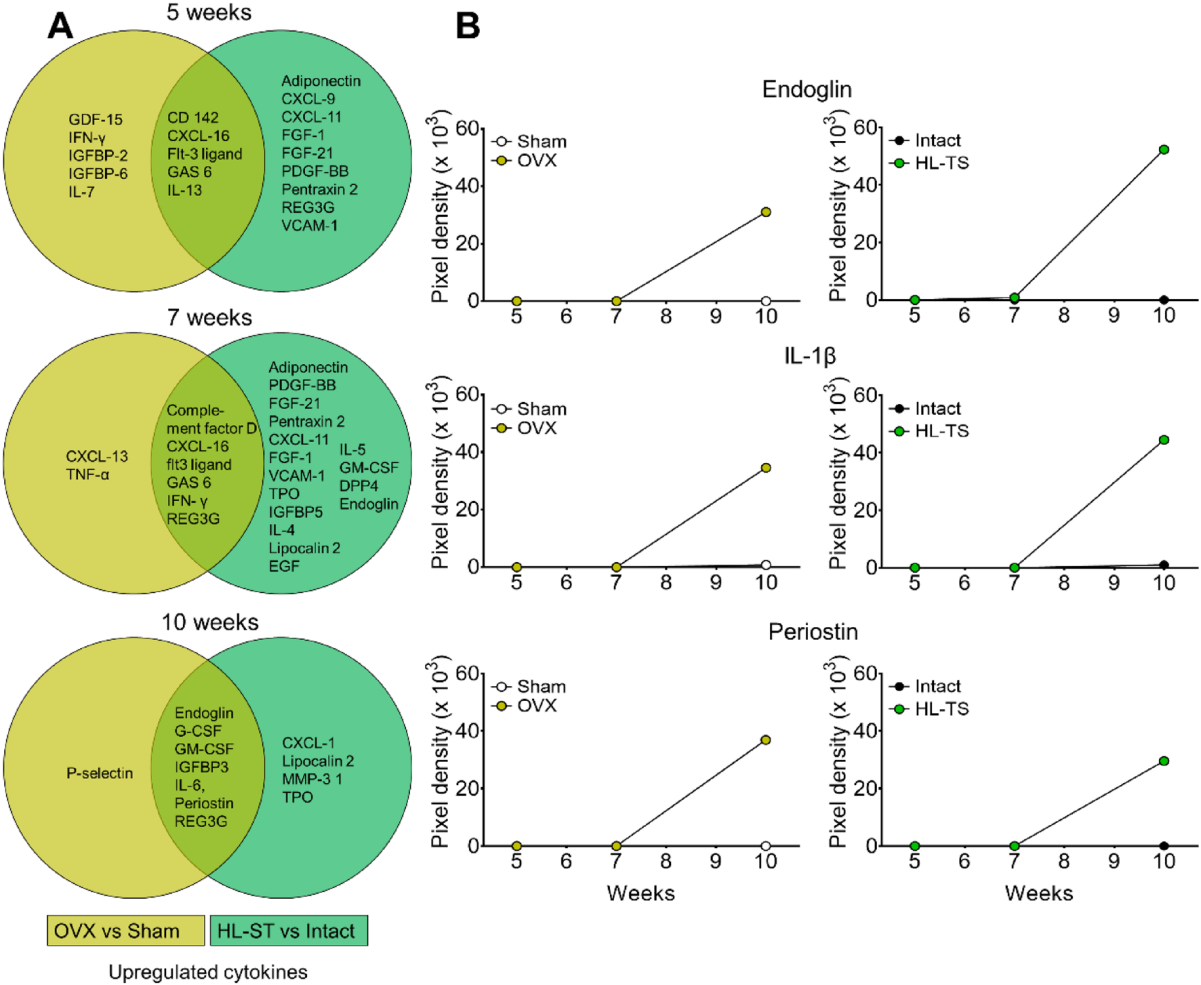
Comparison of cytokines content in circulating EVs. **A** Venn diagrams showing the time-dependent distribution across the groups of the identified differentially up-regulated cytokines in OVX and HL-TS mice compared to their control. Analysis was run on a pool of proteins extracted from 3 animals for each time and groups. No statistics available (for explanation see “Statistics” section in “Methods” section). **B** Time-dependent quantification of pixel density (Arbitrary Units) of the indicated cytokines in circulating EVs from OVX and HL-TS mice and their controls. Graphs are the values of a pool of 3 animals for each time and groups. No statistics available (for explanation see “Statistics” section in “Methods” section)

**Fig. 5:**
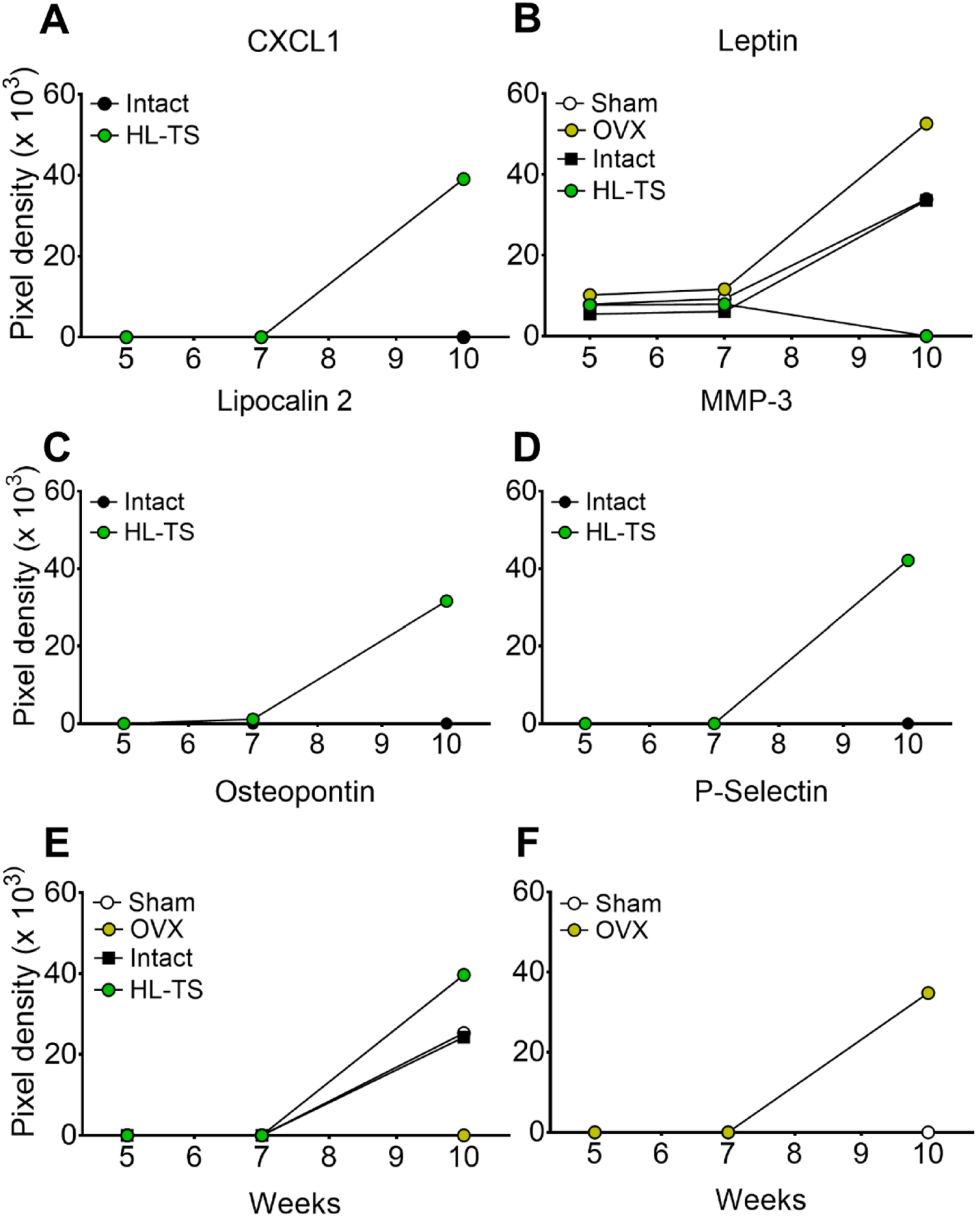
Cytokines with a unique profile in the circulating EVs from OVX and HL-TS mice. **A–F** Time-dependent quantification of pixel density of the indicated cytokines in circulating EVs from OVX and HL-TS mice versus their controls. Graphs are the values of a pool of 3 animals for each time and groups. No statistics available (for explanation see “Statistics” section in “Methods” section)

Bioinformatics analysis revealed sets of proteins common or unique for OVX versus sham-operated mouse serum EVs and for HL-TS versus intact mouse EVs at each timepoint (Fig. 3, Table 3). Among the shared protein, interestingly the kinetics of Endoglin, IL-1β and Periostin were similar in the serum EVs of OVX and HL-TS groups of mice at 10-week timepoint, while they remained undetectable or poorly expressed in control groups (Fig. 4). Instead, a combination of six cytokines appeared to be distinctive overtime for OVX or HL-TS conditions compared to control groups, with diverse or even opposite expression profiles (Table 3, Fig. 5). In this context, **CXCL1** was detectable only at late stage of HL-TS (Fig. 5a), while it was never detectable in OVX or control groups at any time. **Leptin** was found in all groups at early and middle times, but at late time this protein increased in OVX, while in the HL-TS group, it dramatically dropped to undetectable level (Fig. 5b). **Lipocalin 2** was exclusively expressed overtime in serum EVs from HL-TS mice (Fig. 5c), while it was never detected in OVX mice and control groups. Similarly, **Metalloproteinase 3** (MMP-3) increased in late stage of HL-TS (Fig. 5d), while it was never detected in the other groups. **Osteopontin** was undetectable in the OVX group overtime, while it arose at late stage in control groups and even more in HL-TS (Fig. 5e). Finally, **P-Selectin** was only detectable in late stage OVX mouse serum EVs (Fig. 5f), whereas it was undetectable in the other groups overtime.

**Table 3:** Cytokines uniquely up-regulated or down-regulated in circulating EVs from OVX and HL-TS mice using the Mouse XL Cytokine Array Kit (#ARY028, R&D Systems)

Weeks		
5^a^	7^a^	10^a^
Protein	Protein	Protein
*OVX versus Sham-operated mice*		
Up-regulated proteins (not detectable in Sham-operated mice)		
CD 142	FLT3 Ligand	Endoglin
CXCL-16	GAS 6	IGFBP3
FLT3 Ligand	IFN-γ	IL-6
GAS 6	CXCL-16	G-CSF
GDF-15	REG3G	GM-CSF
IFN-γ	CXCL-13	P-Selectin
IGFBP-2	Complement factor D	Periostin
IGFBP-6	TNF-α	
IL-7		
IL-13		
Down-regulated proteins (not detectable in OVX)		
		CCL 21
		Osteopontin
		TNF-α
*HL-TS versus intact mice*		
Up-regulated proteins (not detectable in Intact mice)		
Adiponectin	Adiponectin	CXCL1
CD 142	Complement factor D	Endoglin
CXCL9	CXCL11	G-CSF
CXCL11	CXCL-16	GM-CSF
CXCL-16	DPP4	IGFBP3
FGF-1	EGF	IL-6
FGF-21	Endoglin	Lipocalin 2
FLT3 ligand	FGF-1	MMP-3
GAS 6	FGF-21	Periostin
IL-13	FLT3 Ligand	
PDGF-BB	GAS 6	
Pentraxin 2	GM-CSF	
REG3G	IFN-γ	
VCAM-1	IGFBP5	
	IL-4	
	IL-5	
	Lipocalin 2	
	PDGF-BB	
	Pentraxin 2	
	REG3G	
	Thrombopoietin	
	VCAM-1	
Down-regulated proteins (not detectable in HL-TS)		
		Leptin

^a^Weeks from the start of the experiment

**Fig. 6:**
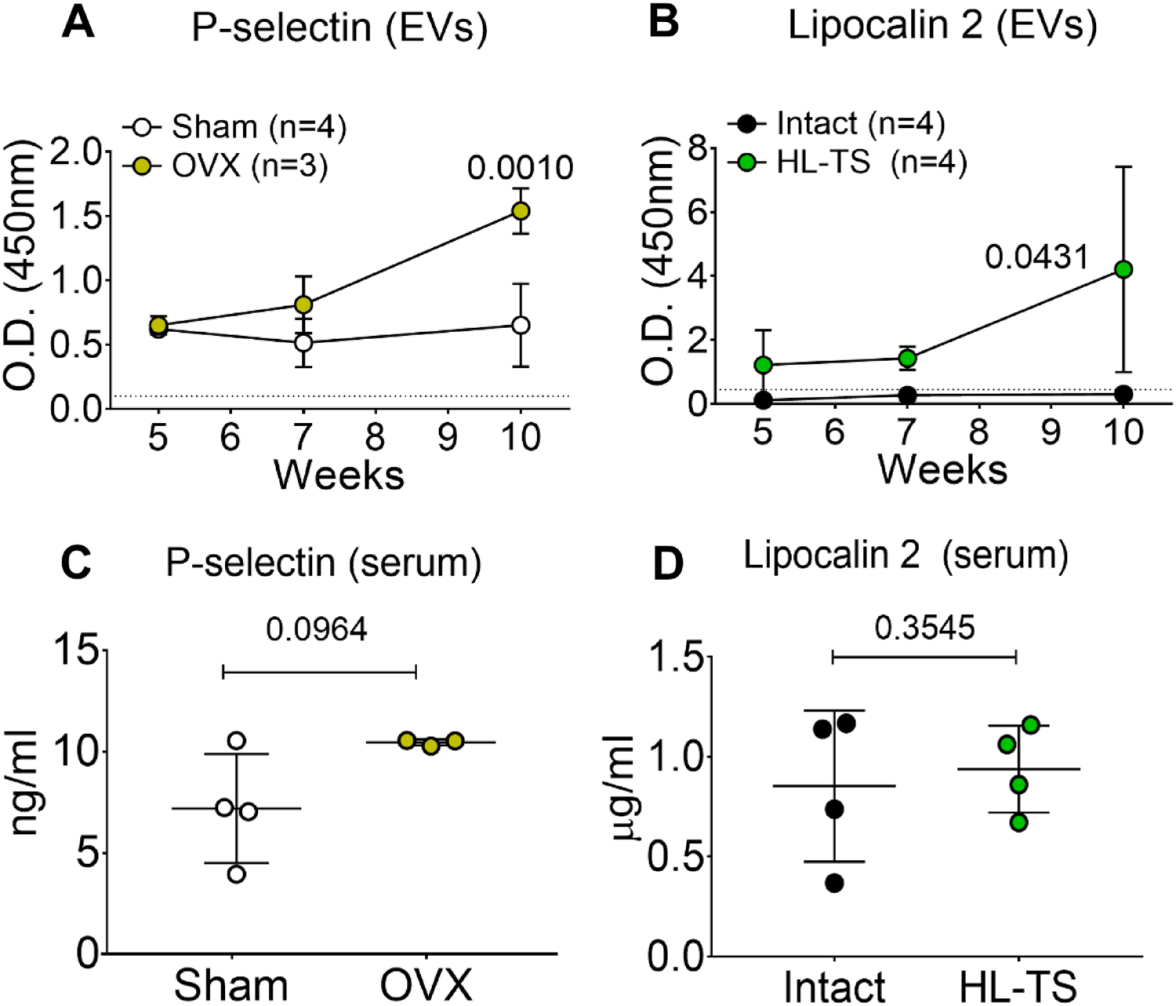
Quantification of P-Selectin and Lipocalin 2 in circulating EVs and sera. **A** Time-dependent ELISA assay for P-Selectin and **B** Lipocalin 2 in protein lysates from circulating EVs of OVX, HL-TS and control mice. **C** ELISA assay for P-Selectin and **D** Lipocalin 2 in the sera from of OVX, HL-TS and control mice at the end of the experiments. The number of mice is shown in the figure panels and were selected by randomization. Statistics: in (**A**, **B**) one-way ANOVA using non-parametric Dunnet post hoc test among multiple groups (whole curves OVX vs. Sham and HL-TS vs. Intact mice). In (**C**, **D**) Student’s *t* test. *p* values are shown in the graphs

**Fig. 7:**
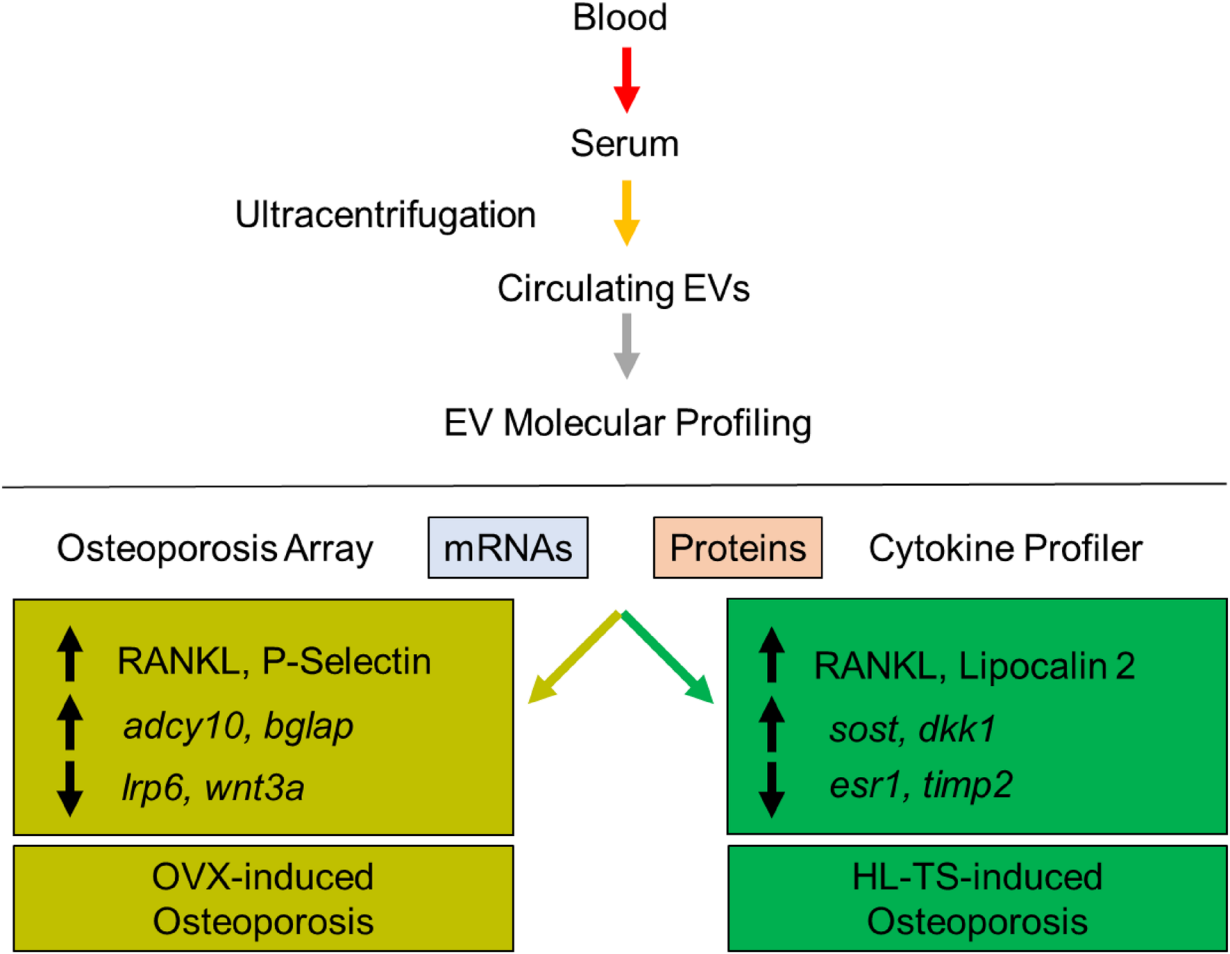
Scheme summarising the results, highlighting the differential molecular profiles of EVs circulating in OVX and HL-TS mice. mRNAs and cytokine changes investigated are limited to those present in the mouse Osteoporosis PCR array (#PAMM‐170Z, RT^2^ Profiler Real-Time PCR Array, Qiagen) and in the Mouse XL Cytokine Array Kit (#ARY028, R&D Systems), respectively

To validate the results with a standard statistically reliable method, we focused on P-selectin and Lipocalin 2, analysing their concentration in circulating EVs and whole sera of control and osteoporotic mice by ELISA. P-selectin remained at steady concentration in circulating EVs of control mice, while its expression progressively increased in the circulating EVs of OVX mice (Fig. 6a). Lipocalin 2 was barely detectable in control circulating EVs while increased at late stage in HL-TS mice (Fig. 6b). Interestingly, ELISA performed in the mouse sera at the end of the experiment (10 weeks after the induction of osteoporosis) did not detect differences in soluble P-selectin and Lipocalin 2 in the osteoporosis groups versus their controls (Fig. 6c, d). This observation may provide circulating EVs, rather than the sera themselves, a unique differential diagnostic meaning in conditions mimicking postmenopausal or disuse osteoporosis. The summary of the results is depicted in Fig. 7.

## Discussion

In this work, we provided evidence that circulating EVs may have the potential to be used as reliable means to monitor the development of osteoporosis and to serve as differential diagnostic biomarker tools for post-menopausal and disuse osteoporosis.

We demonstrated that primary murine osteoblasts release surface RANKL-expressing EVs in an inducible manner upon steroid depletion and microgravity. These results are in line with our previous observations demonstrating that the treatment with parathyroid hormone, another regulator of bone metabolism and RANKL inducer, prompts an increase of RANKL-positive EVs compared to untreated control [37]. The same results were confirmed in vivo in circulating EVs in our mouse models of post-menopausal and disuse osteoporosis. Interestingly, RANKL is shared by various human forms of osteoporosis [16, 26] and, according to our previous results, RANKL-expressing EVs have biological functions, supporting osteoclast survival in vitro and triggering osteoclastogenesis when injected systemically in RANKL-null mice [37], indirectly suggesting that circulating RANKL-positive EVs could also represent biological tools contributing to exacerbated osteoclastogenesis in osteopenic diseases.

The differences were not limited to the RANKL profile but involved both the transcriptome and the proteome. To address the transcriptomic differences in the serum EVs, we focused on genes associated with bone metabolism, preventing confounding information by bone-unrelated factors carried by the circulating EV pool. Many EV mRNAs were regulated overtime during the progression of osteoporosis in our mice, including genes encoding proteins involved in osteoclast (*Acp5, Adcy10, Calca, Ctsk, Pth, Il-6, Tnfsf11*, *Vegfa*) [41–43] and osteoblast (*Dkk1, Enpp1, Il-6, Lrp6, Wnt3a)* [44, 45] formation, regulation and activity, as well as in bone matrix assembly and mineralization (*Alpl, Bglap, Col1a1)* [46] and in coupling bone resorption with bone formation (*Tgfb1*, *Ltbp2)* [47]*.* Interestingly, in OVX mice, circulating EVs carried a unique panel of mRNAs involved in osteoblast function, such as *Bglap, Col1a1, Dkk1* and *Lrp6*, while mRNAs involved in osteocyte function, such as *Sost* and *Timp2*, were uniquely noted in HL-TS circulating EVs.

A protein profile shift was also detected in osteoporotic versus control circulating EVs, in part shared by OVX and HL-TS mice. Evaluation of **RANKL** by flow cytometry showed more RANKL-positive EVs in both osteoporotic conditions, albeit with non-overlapping kinetics, which was transient in OVX and sustained in HL-TS mice. Similar kinetics were instead shown in the two conditions by Endoglin, IL-1β and Periostin. **Endoglin** is an accessory receptor for TGF-β playing a role in the regulation of fibrosis and angiogenesis [48] and in osteoblastic differentiation and bone matrix mineralization [49]. **IL-1β** affects both osteoblastogenesis [50] and osteoclastogenesis [51–53] and was described in the onset of OVX- and unloading/disuse-induced osteoporosis [54–57]. **Periostin** is a matricellular protein binding integrins αvβ3 and αvβ5 regulating cell adhesion and mobility [58] and inducing β-catenin regulated pathways [59]. Periostin is also known to be associated with bone loss [60].

A group of proteins were instead differentially modulated in circulating EVs from OVX and HL-TS mice. EVs from HL-TS mice expressed uniquely CXCL1, Lipocalin 2 and MMP-3, while OVX-derived circulating EVs were exclusively enriched in P-Selectin. **CXCL1** has effects on neutrophils, endothelial cells and epithelial cells and is associated with tissue damage and diseases [61–63]. It is involved in skeletal muscle fitness, especially in sarcopenic models, in which overexpression of CXCL1 was found in necrotic fibres and in blood vessels of damaged muscles [64, 65]. This is in line with our results, given that HL-TS also induces muscle wasting [66]. **Lipocalin 2** is a pleiotropic cytokine involved in many processes. Our group demonstrated both in vitro and in vivo that Lipocalin 2 plays a pivotal role in mechanical unloading-related processes [22], while it appears to have no implication in OVX-induced osteoporosis [15]. **MMP-3** is a proteinase involved in the degradation of fibronectin, laminin, elastin, collagen IV and proteoglycans [67, 68] that exerts a pro-inflammatory action [69]. MMP-3 is reported to be associated with bone resorption [70], fat mass [71] and muscle wasting caused by nerve fibre demyelination [72] and multiple sclerosis [73]. The direct association reported between the onset of muscle disease and MMP-3 levels seems in line with our findings, appearing specifically only in late stage of disuse-induced HL-TS. **P-Selectin** is released by endothelial cells and activated platelets and plays an essential role in the initial recruitment of leukocytes and in the amplification of the immune reaction [74]. In agreement with this function, we found P-selectin-positive EVs in OVX-induced osteoporosis, in which the involvement of leukocyte activation and inflammation is well elucidated [21]. Finally, the kinetics of **Leptin** and **Osteopontin** were opposite in the two forms of osteoporosis, with Leptin increasing in OVX and declining in HL-TS, and Osteopontin increasing in HL-TS but not in OVX. These results are consistent with the knowledge that Leptin stimulates [75] and Osteopontin inhibits [76] osteoblast function, thus supporting the known different contribution of this cell type in the two pathological conditions [77, 78].

This study could not allow the identification of neither the cellular sources of circulating EVs nor the potential contamination with matrix vesicles typically involved in matrix mineralization. However, it appears evident that osteoblast molecules are represented in our EV pool and, taken together, the results unveiled molecular differences in EVs that are likely to represent differential signatures of the two osteoporotic conditions tested. These signatures converge on pathways associated with the increase of osteoclast function, such as the RANKL pathway, shared by EVs circulating in both models of osteoporosis, but diverge in pathways, such as Leptin, Lipocalin 2 and Osteopontin, whose modulations are consistent with impairment of osteoblast function and are apparently more prominent in HL-TS, likely disturbing the balance of the bone multicellular unit in a manner different from OVX. We believe that these results pave the way to clinical studies for the use of circulating EVs as new potent tool for the diagnosis and the monitoring of the response to therapy in human osteoporosis with different aetiology.

## Supplementary Information

Below is the link to the electronic supplementary material.

Supplementary file 1 (DOCX 2842 KB)
