# Supplementary material for: Circulating Extracellular Vesicles Express Receptor Activator of Nuclear Factor κB Ligand and Other Molecules Informative of the Bone Metabolic Status of Mouse Models of Experimentally Induced Osteoporosis
Source: Calcif Tissue Int. 2022 Oct 25;112(1):74–91. doi: 10.1007/s00223-022-01032-5 (PMC9813163; doi:10.1007/s00223-022-01032-5)
Supplement: Supplementary file 1 — (DOCX 2842 KB) [file 223_2022_1032_MOESM1_ESM.docx]

**Title: Circulating extracellular vesicles express Receptor Activator of Nuclear Factor κ β**

**Ligand (RANKL) and other molecules informative of the bone metabolic status of mouse models of experimentally induced osteoporosis.**

**Corresponding Author**: Anna Teti, [annamaria.teti@univaq.it](mailto:annamaria.teti@univaq.it)

**Supplementary Table 1 - The ARRIVE Checklist**

In agreement with the ARRIVE guidelines, we provide the ARRIVE checklist with the scope to achieve reproducibility for the manuscript reporting results of experiments involving animals by listing the kinds of information needed to ensure that this manuscript contain the rationale and limitations underlying the experimental and statistical approach, and the information needed to reproduce the reported experiments and comparison with previous studies. This checklist is mean to aid the authors in providing detailed information within this submission that meets a standard to achieve reproducibility and transparency of research, and assists reviewers in their effort determine whether the necessary information is present.

|  | | ITEM | RECOMMENDATION | Section/ Paragraph | |
| --- | --- | --- | --- | --- | --- |
| 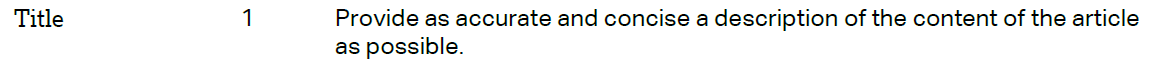 | | | Pag 1 | |  |
| 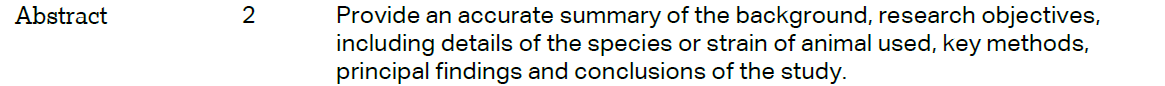 | | | Pag 2 | |  |
| INTRODUCTION | | |  | |  |
| 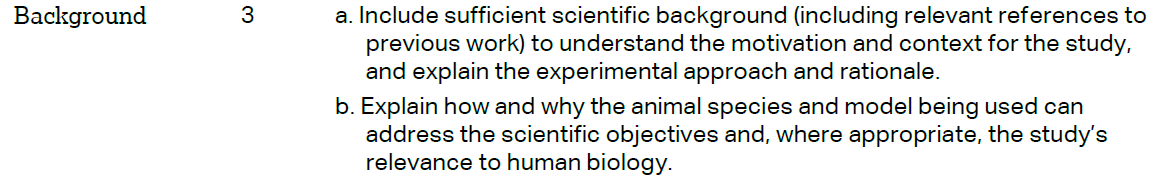 | | | Pag 4-6 | |  |
| 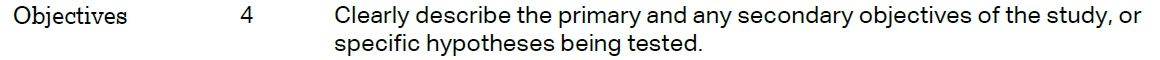 | | | Pag 5-6 | |  |
| METHODS | | |  | |  |
| 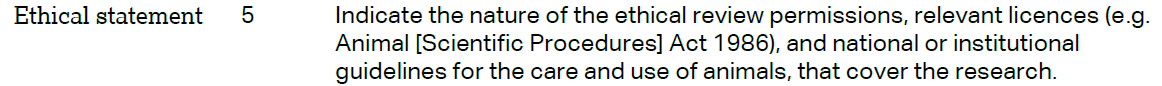 | | | Pag 6 | |  |
| 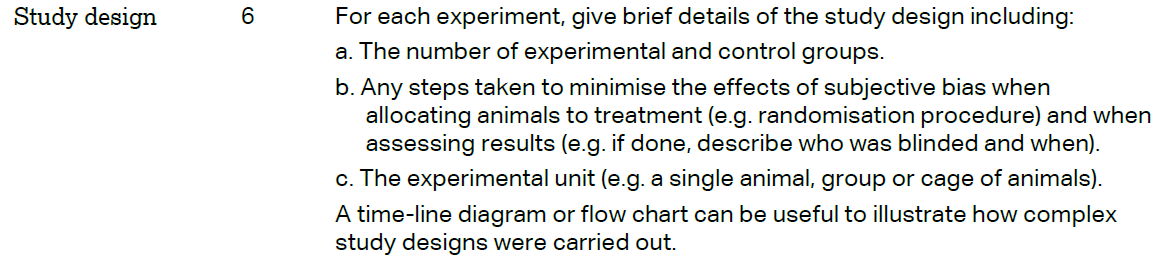 | | | Pag 6 | |  |
| 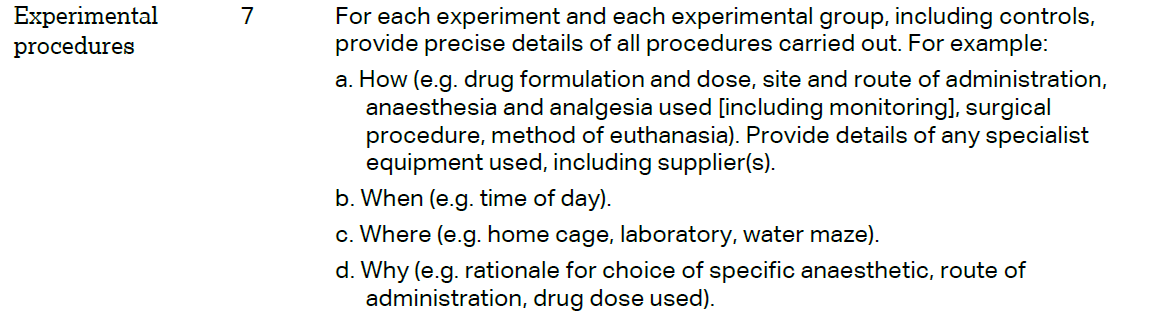 | | | Pag 6 | |  |
| 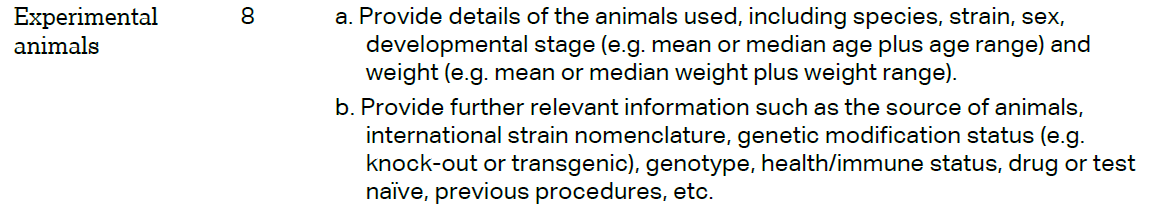 | | | Pag 6 | |  |
| 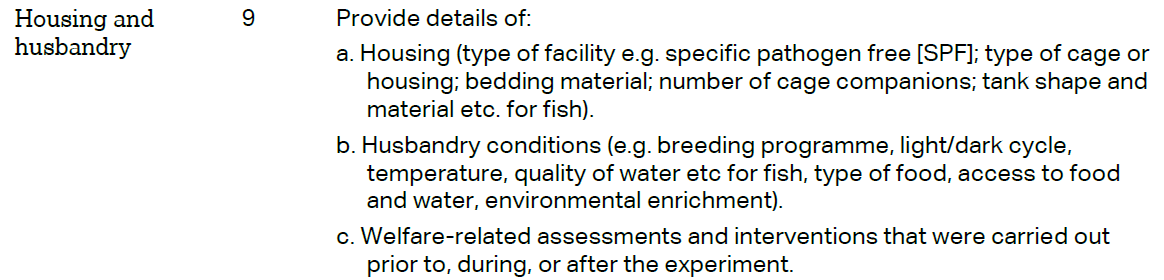 | | | Pag 6 | | |
| 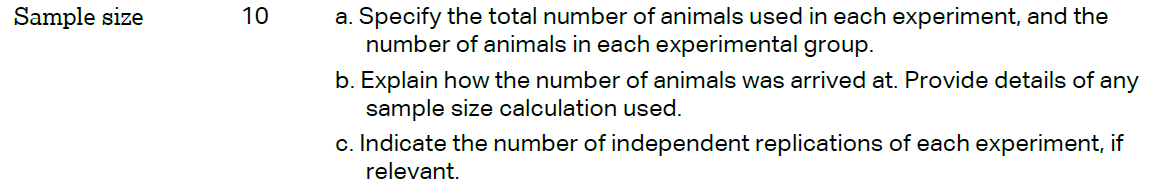 | | | Pag 6 | | |
| 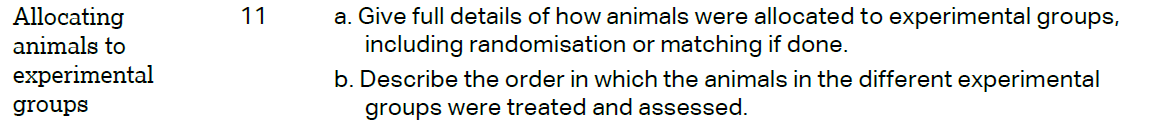 | | | Pag 6 | | |
| 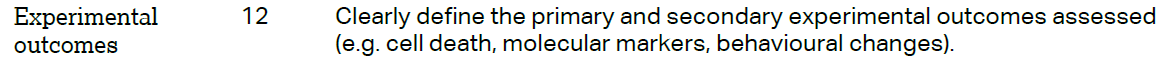 | | | Pag 6 | | |
| 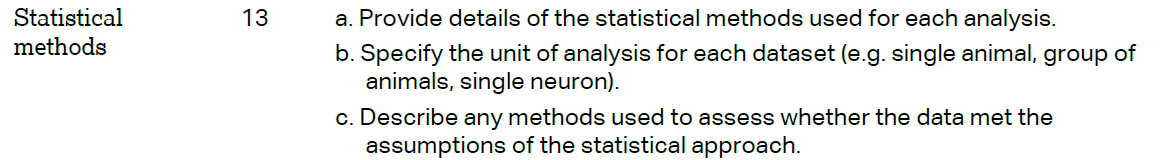 | | | Pag 12 | | |
| RESULTS | | |  | | |
| 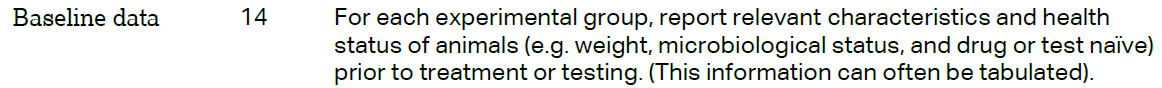 | | | Pag 6 | | |
| 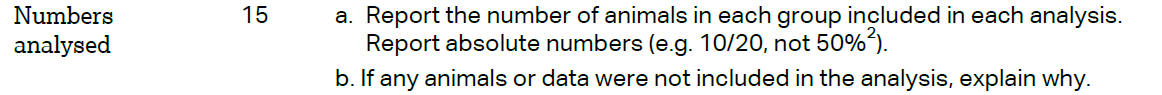 | | | Pag 6 | | |
| 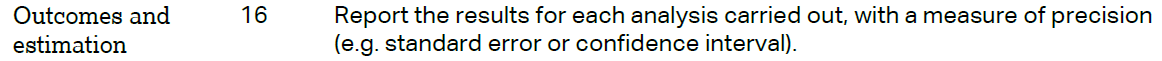 | | | Figures 2-6, supplementary figures S1-S4 | | |
| 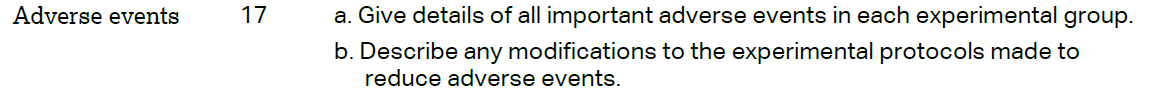 | | | Pag 6 | | |
| DISCUSSION | | |  | | |
| 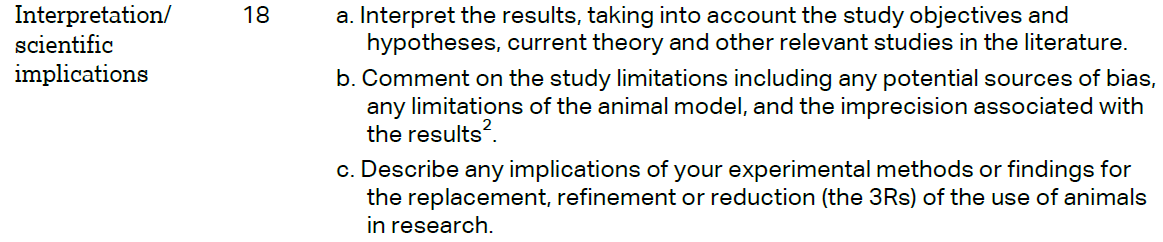 | | | Pag 11-19 | | |
| 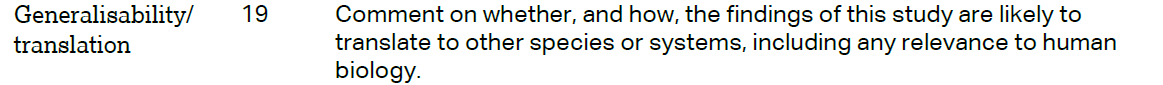 | | | Pag 11-19 | | |
| 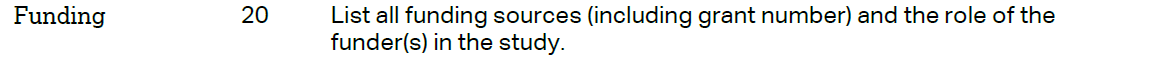 | | | | Pag 20 |  |


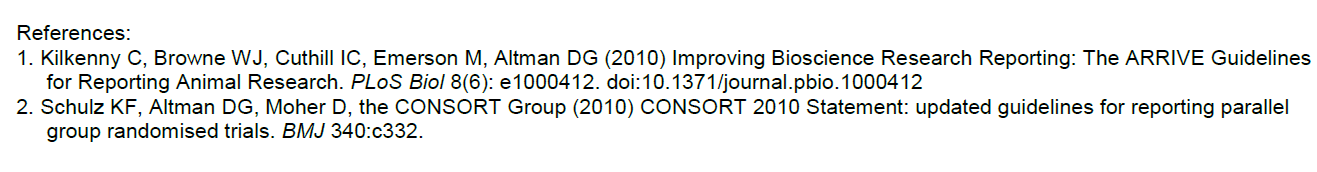


**Supplementary Table 2 – List of primers used for RT-PCR**

| **Gene** | **Forward** | **Reverse** |
| --- | --- | --- |
| *col1a1* | 5′- GTCCCTCTGGAAATGCTGGAC-3′ | 5′-GACCGGGAAGACCGACCA-3′ |
| *gapdh* | 5′-TGGCAAAGTGGAGATTGTTGC‐3′ | 5′‐AAGATGGTGATGGGCTTCCCG‐3′ |
| *il1-β* | 5’-ATGGCAACTGTTCCTGAACTCAAGT-3’ | 5’-CAGGACAGGTATAGATTCTTTCCTT-3’ |
| *lcn2* | 5′‐CCAGTTCGCCATGGTATTTT‐3′ | 5′‐CACACTCACCACCCATTCAG‐3′ |
| *tnfsf11* | 5′‐CCAAGATCTCTAACATGACG‐3′ | 5′‐CACCATCAGCTGAAGATAGT‐3′ |

**Supplementary Table 3 – Genes expressed in circulating EVs from control, OVX and HL-TS mice** using the mouse Osteoporosis PCR array (#PAMM‐170Z, RT^2^ Profiler Real Time PCR Array, Qiagen).

| **Gene** | **Intact mice** | | | | | | **Sham-operated mice** | | | | | | **HL-TS mice** | | | | | | **OVX mice** | | | | | |
| --- | --- | --- | --- | --- | --- | --- | --- | --- | --- | --- | --- | --- | --- | --- | --- | --- | --- | --- | --- | --- | --- | --- | --- | --- |
|  | **Weeks*** | | | | | | **Weeks*** | | | | | | **Weeks*** | | | | | | **Weeks*** | | | | | |
|  | **5** | | **7** | | **10** | | **5** | | **7** | | **10** | | **5** |  | **7** |  | **10** | | **5** | | **7** | | **10** | |
|  | **Ct** | **SD** | **Ct** | **SD** | **Ct** | **SD** | **Ct** | **SD** | **Ct** | **SD** | **Ct** | **SD** | **Ct** | **SD** | **Ct** | **SD** | **Ct** | **SD** | **Ct** | **SD** | **Ct** | **SD** | **Ct** | **SD** |
| *Acp5* | 31.44 | 2.33 | 35.07 | 0.99 | 34.59 | 2.22 | 31.29 | 2.63 | 35.64 | 0.75 | 34.59 | 2.22 | 23.50 | 0.42 | 34.12 | 0.06 | 30.09 | 0.49 | 25.57 | 1.22 | 30.11 | 0.56 | 26.34 | 0.38 |
| *Actb* | 31.12 | 5.73 | 23.11 | 1.47 | 25.64 | 4.95 | 32.49 | 6.41 | 23.68 | 1.11 | ND | ND | 30.10 | 2.34 | 23.47 | 0.87 | 24.99 | 3.63 | 27.91 | 3.65 | 25.35 | 0.73 | 28.27 | ND |
| *Adcy10* | 36.18 | 1.27 | 37.16 | 3.93 | 32.66 | ND | 35.78 | 3.68 | 34.60 | ND | ND | ND | 33.10 | 1.61 | 29.21 | ND | 26.91 | 12.71 | 21.80 | 0.62 | ND | ND | 31.30 | 5.26 |
| *Alox12* | 34.89 | 2.71 | 30.98 | ND | ND | ND | 33.55 | 0.28 | 31.15 | ND | 37.61 | 2.91 | 32.89 | 3.70 | 27.54 | 0.54 | 29.84 | 4.40 | 21.81 | 7.00 | 33.30 | 4.52 | 35.70 | ND |
| *Alox15* | ND | ND | 30.93 | ND | 36.88 | 3.86 | ND | ND | 31.10 | ND | 28.28 | 0.97 | 36.42 | 3.35 | ND | ND | 37.59 | 0.08 | 25.92 | 2.76 | 38.31 | ND | 30.98 | 2.91 |
| *Alox5* | 33.19 | 1.87 | 32.19 | 0.29 | 30.78 | 3.96 | 34.40 | 2.04 | 32.91 | 0.47 | 27.17 | 0.10 | 34.35 | 2.65 | ND | ND | 27.11 | 6.97 | 30.22 | 2.37 | 38.27 | 1.53 | 28.94 | 0.04 |
| *Alpl* | 29.31 | 2.75 | 29.26 | 0.72 | 27.73 | 0.96 | 27.61 | 1.12 | 29.86 | 0.47 | 31.35 | ND | 28.82 | 2.09 | 29.05 | 0.69 | 36.67 | 0.37 | 25.39 | 0.14 | 29.41 | 1.23 | 28.15 | ND |
| *Ar* | 33.92 | 1.80 | ND | ND | 34.55 | 4.75 | 32.12 | 6.26 | ND | ND | 27.02 | 0.35 | 34.90 | 2.23 | ND | ND | ND | ND | 24.86 | 0.12 | 28.33 | ND | 29.11 | 1.93 |
| *B2m* | 27.77 | 3.88 | 24.18 | 1.16 | 23.38 | 2.96 | 28.78 | 4.05 | 24.73 | 0.86 | 36.81 | 0.08 | 26.71 | 1.41 | 26.09 | 3.53 | 24.73 | 0.27 | 32.97 | 0.25 | 25.98 | 1.57 | 33.96 | 0.06 |
| *Bglap* | 32.18 | 2.07 | 31.97 | 2.44 | 27.02 | 0.30 | 29.64 | 0.26 | 32.58 | 2.09 | 35.62 | 3.61 | 33.24 | 1.12 | 30.28 | 0.17 | 28.39 | 4.18 | 24.51 | 0.21 | 32.91 | 1.89 | 33.47 | ND |
| *Bmp2* | 35.17 | 2.64 | 35.99 | 0.27 | 36.93 | 0.01 | 28.69 | 2.52 | 36.68 | 0.61 | 30.21 | 0.88 | 27.73 | 9.67 | 35.59 | ND | 35.02 | 2.58 | 30.01 | 0.48 | 35.78 | 3.52 | 31.16 | 4.44 |
| *Bmp7* | 32.70 | 1.84 | 36.78 | 2.12 | 36.67 | 3.13 | 32.19 | 1.26 | 37.38 | 2.55 | 29.25 | 0.61 | 32.63 | 2.63 | 38.72 | ND | 22.42 | ND | 24.66 | 0.12 | 31.73 | 2.09 | 30.35 | 2.74 |
| *Calca* | 31.71 | 1.56 | 35.15 | 2.78 | 31.73 | 2.88 | 31.64 | 0.21 | 35.78 | 2.44 | 20.15 | 1.18 | 29.64 | 6.37 | 31.81 | 3.44 | 28.30 | 7.38 | ND | ND | 36.75 | 0.63 | 27.32 | 1.82 |
| *Calcr* | 30.39 | 1.76 | 30.98 | 0.15 | 30.40 | 2.30 | 29.76 | 2.27 | 31.52 | 0.37 | 35.28 | 2.74 | 30.31 | 2.01 | 30.57 | 4.56 | 26.08 | 5.03 | 27.53 | 0.15 | 30.87 | 0.54 | 33.33 | 4.20 |
| *Car2* | 28.20 | 1.22 | 25.52 | 0.57 | 22.91 | 5.57 | 30.25 | 3.62 | 26.13 | 0.39 | 26.73 | ND | 32.92 | 4.78 | 25.00 | 3.95 | 32.53 | 2.26 | ND | ND | 31.98 | ND | ND | ND |
| *Casr* | 28.37 | 1.20 | 35.07 | 0.28 | 33.23 | 3.15 | 28.77 | 2.26 | 35.76 | 0.69 | 32.72 | 4.35 | 26.45 | 5.38 | 31.88 | 4.94 | 28.40 | 7.71 | 30.05 | 1.04 | 32.08 | 1.65 | 30.77 | 1.84 |
| *Cd40* | 35.54 | 2.86 | 39.69 | 0.33 | 26.61 | ND | 34.06 | 0.33 | ND | ND | 34.45 | 5.06 | 35.94 | 3.10 | ND | ND | 27.73 | 7.80 | 31.54 | 2.31 | 31.49 | ND | 33.41 | 1.72 |
| *Clcn7* | 29.67 | 0.67 | 36.08 | 1.67 | 31.57 | 3.98 | 29.86 | 1.18 | 36.65 | 2.04 | 27.71 | 0.35 | 30.38 | 1.93 | 34.91 | ND | 30.65 | 9.28 | 30.87 | 3.72 | 35.25 | 3.59 | 23.56 | 0.03 |
| *Cnr2* | 33.08 | 0.97 | 39.25 | 0.74 | 34.13 | 4.97 | 32.05 | 1.75 | 38.91 | ND | 25.46 | 3.90 | 33.44 | 1.72 | 37.61 | ND | 35.61 | 3.46 | 32.74 | 4.36 | 35.57 | 0.82 | 24.74 | 4.96 |
| *Col1a1* | 29.55 | 1.03 | 27.00 | 1.79 | 29.14 | 1.62 | 28.90 | 0.91 | 27.52 | 1.48 | 33.90 | 5.97 | 28.64 | 1.35 | 25.62 | 0.10 | 26.04 | 5.67 | 30.77 | 1.75 | 26.57 | 1.12 | 30.80 | 4.97 |
| *Col1a2* | 32.24 | 4.48 | 26.10 | 1.30 | 25.86 | 4.41 | 33.71 | 4.51 | 26.66 | 1.01 | 31.32 | 3.19 | 34.75 | 2.75 | 25.87 | 1.31 | 24.47 | 3.30 | 26.08 | 6.20 | 27.41 | 1.24 | 29.12 | 3.97 |
| *Comt* | 36.78 | 1.54 | 29.00 | 0.36 | 32.12 | 7.42 | 24.19 | 8.97 | 29.78 | 0.48 | 30.01 | ND | 34.87 | ND | 37.62 | ND | 30.44 | 2.39 | 20.81 | 0.12 | 32.81 | 6.72 | 25.94 | 0.31 |
| *Crtap* | 33.10 | 1.33 | 39.48 | 0.59 | 32.31 | 4.38 | 25.82 | 6.49 | ND | ND | 35.42 | 2.27 | 32.62 | 1.97 | 29.47 | 3.16 | 29.79 | 8.78 | 24.46 | 0.12 | 36.28 | 1.71 | 32.99 | 0.91 |
| *Ctsk* | ND | ND | 38.44 | 0.50 | 29.90 | ND | 37.29 | 2.52 | 35.86 | 5.84 | ND | ND | ND | ND | 37.72 | 0.55 | 27.78 | 8.53 | 30.99 | 0.12 | 29.87 | ND | ND | ND |
| *Cyp17a1* | ND | ND | ND | ND | 35.62 | 2.83 | ND | ND | ND | ND | 33.25 | 5.56 | 20.53 | ND | ND | ND | 36.84 | ND | 25.75 | 11.23 | ND | ND | 31.22 | 8.17 |
| *Cyp19a1* | 36.43 | ND | ND | ND | ND | ND | 28.56 | 10.23 | ND | ND | 33.52 | ND | 32.86 | 4.71 | 29.01 | ND | 30.96 | 5.59 | 39.63 | 0.12 | 34.13 | ND | ND | ND |
| *Dbp* | 33.25 | 2.24 | 30.11 | 0.40 | 33.24 | 5.68 | 27.84 | 6.08 | 30.90 | 0.55 | 30.79 | 3.05 | 33.87 | 1.97 | 37.59 | 2.01 | 23.03 | 1.46 | 25.93 | 7.22 | 33.65 | 7.59 | 30.22 | 1.14 |
| *Dkk1* | ND | ND | ND | ND | 33.40 | ND | ND | ND | ND | ND | 31.64 | 3.77 | ND | ND | 28.87 | ND | 31.61 | 0.03 | 29.65 | 5.26 | 31.11 | ND | 27.66 | 3.28 |
| *Enpp1* | 29.82 | 1.47 | 29.65 | 1.42 | 30.45 | 2.94 | 28.93 | 2.91 | 30.29 | 1.04 | ND | ND | 30.80 | 1.76 | 37.59 | 0.90 | 28.25 | 3.79 | 35.35 | 4.60 | 37.29 | 1.23 | 28.55 | 10.39 |
| *Esr1* | 33.56 | ND | 34.52 | 1.18 | 33.21 | 5.75 | 30.01 | 6.72 | 35.08 | 0.85 | 25.84 | ND | 39.00 | 0.32 | 38.22 | 0.84 | 34.25 | 3.93 | 27.83 | 9.67 | 36.71 | 2.61 | ND | ND |
| *Esr2* | 38.94 | ND | 33.70 | ND | ND | ND | 39.63 | ND | 33.82 | ND | 25.86 | ND | 39.30 | ND | ND | ND | 33.84 | 4.41 | 23.31 | 0.12 | ND | ND | 26.40 | ND |
| *Esrra* | 38.36 | ND | 36.47 | ND | 31.50 | 8.17 | 35.55 | 1.76 | 36.63 | ND | 29.25 | ND | 36.91 | 1.75 | 27.57 | ND | 32.34 | 0.58 | ND | ND | 38.52 | ND | 27.45 | ND |
| *Fgfr1* | 32.11 | 1.45 | 37.26 | 1.52 | 29.91 | 5.86 | 32.64 | 3.74 | 37.31 | 2.25 | ND | ND | 32.58 | 2.00 | 33.73 | 2.93 | 26.26 | 1.02 | 36.84 | 0.12 | 29.38 | 2.99 | 39.16 | ND |
| *Fgfr2* | 33.16 | 1.58 | 36.43 | 1.25 | 32.74 | 5.08 | 25.83 | 4.25 | 37.05 | 1.66 | 32.97 | 2.32 | 33.18 | 3.81 | 24.82 | ND | 37.16 | 3.27 | 31.41 | 6.52 | 38.57 | 0.45 | 30.37 | 3.06 |
| *Gapdh* | 22.74 | 0.11 | 26.84 | 0.23 | 23.95 | 0.70 | 22.64 | 0.43 | 27.46 | 0.50 | 29.24 | 4.36 | 22.25 | 0.28 | 27.42 | 0.28 | 24.28 | 0.41 | 22.49 | 0.41 | 27.83 | 0.12 | 28.20 | 5.82 |
| *Ghrh* | 33.34 | 1.64 | 36.97 | ND | 36.80 | ND | 28.96 | 0.48 | 37.12 | ND | 28.52 | 0.90 | 32.32 | 3.43 | 38.95 | ND | 28.65 | 5.02 | 31.79 | 5.83 | 36.17 | 3.71 | 29.40 | 1.96 |
| *Gusb* | 31.26 | 0.68 | 38.16 | ND | 29.74 | 5.05 | 31.45 | 2.11 | 38.36 | ND | 30.13 | 0.81 | 33.23 | 2.46 | 35.94 | 1.99 | 17.60 | ND | 21.77 | 5.48 | 35.50 | 4.26 | 32.49 | 5.32 |
| *Hsd11b1* | 35.71 | 3.21 | 39.13 | ND | 31.97 | 3.44 | 26.91 | 6.25 | 39.38 | ND | 34.81 | 0.11 | 35.36 | 2.23 | 30.71 | ND | 32.31 | 5.76 | 23.57 | 6.82 | 37.79 | 1.71 | 27.43 | 0.03 |
| *Hsp90ab1* | 30.00 | 1.04 | 25.57 | 1.01 | 24.45 | 6.90 | 28.19 | 2.12 | 26.15 | 0.72 | 28.81 | 2.38 | 30.00 | 2.73 | 25.00 | 0.46 | 24.58 | 6.33 | 26.61 | 1.64 | 27.29 | 0.93 | 29.83 | 3.82 |
| *Igf1* | 31.42 | 3.59 | 30.76 | 2.25 | 30.32 | 5.50 | 28.98 | 6.65 | 31.43 | 2.69 | 33.42 | ND | 30.47 | 4.83 | 33.53 | 4.22 | 27.69 | 2.25 | 29.82 | 1.29 | 31.01 | 1.92 | 31.87 | 0.19 |
| *Igfbp2* | 32.82 | 1.12 | 35.53 | 3.04 | 29.59 | 2.65 | 26.14 | 6.71 | 36.17 | 3.42 | 27.12 | 2.31 | 31.57 | 0.87 | 29.65 | 3.68 | 25.64 | 6.10 | 21.96 | 5.88 | 33.25 | 3.03 | 29.90 | 6.16 |
| *Il15* | 31.81 | 1.77 | 36.11 | 1.76 | 31.63 | 3.18 | 31.70 | 1.23 | 36.64 | 1.43 | 29.12 | ND | 29.27 | 4.52 | 32.58 | 2.79 | 30.29 | 0.96 | 32.44 | 3.54 | 34.00 | 4.70 | 32.19 | 3.37 |
| *Il6* | 34.38 | 4.64 | ND | ND | 35.86 | 1.86 | 35.01 | 3.97 | ND | ND | 33.73 | 0.13 | 29.60 | 9.44 | 34.70 | 0.24 | 29.16 | 0.03 | 33.16 | ND | 29.16 | ND | 31.56 | 3.29 |
| *Il6ra* | 34.34 | 4.70 | ND | ND | 30.13 | 4.11 | 34.15 | 3.94 | ND | ND | 28.96 | 3.30 | 32.88 | 5.69 | 39.37 | ND | 31.58 | 1.88 | 24.53 | 0.12 | 32.04 | ND | 27.45 | ND |
| *Itga1* | 35.96 | 4.71 | ND | ND | 33.32 | ND | 30.41 | ND | ND | ND | 31.45 | 4.03 | 34.69 | ND | ND | ND | 28.51 | 8.93 | 30.33 | 0.12 | 31.57 | 10.10 | 31.54 | 3.36 |
| *Itgb3* | 30.16 | 1.08 | 27.44 | 0.59 | 28.48 | 4.02 | 28.54 | 1.12 | 28.03 | 1.02 | 25.06 | 1.40 | 30.00 | 2.48 | 29.20 | 4.18 | 29.94 | 3.15 | 27.59 | 0.79 | 29.54 | 0.85 | 29.11 | 1.90 |
| *Lep* | 33.69 | 2.32 | 37.77 | ND | 28.99 | ND | 33.91 | 1.50 | 37.94 | ND | ND | ND | 33.88 | 5.44 | ND | ND | 39.99 | ND | 39.16 | 0.15 | 36.36 | ND | ND | ND |
| *Lrp1* | 36.00 | 2.63 | 27.89 | 0.35 | 28.01 | 4.19 | 27.65 | 9.73 | 28.86 | 0.63 | 26.65 | 1.22 | 28.88 | 2.69 | 23.36 | ND | 33.12 | 5.78 | 18.83 | 0.12 | 33.64 | ND | 29.17 | 0.53 |
| *Lrp5* | 32.13 | 1.61 | 36.83 | 0.38 | 31.60 | 4.31 | 30.54 | 1.22 | 37.51 | 0.48 | 32.67 | ND | 33.09 | 2.80 | 29.04 | ND | 25.35 | 7.91 | 24.66 | 3.51 | 35.63 | 0.96 | 33.98 | 0.42 |
| *Lrp6* | 34.69 | 3.02 | 28.07 | 1.64 | 28.58 | 6.94 | 26.36 | 7.92 | 28.72 | 2.09 | 23.34 | ND | 34.40 | 1.11 | 31.56 | 6.82 | 24.00 | 8.45 | 27.89 | 6.60 | 37.12 | 1.18 | 24.56 | ND |
| *Lta* | 39.00 | ND | ND | ND | 37.32 | ND | 19.81 | 2.35 | ND | ND | 32.76 | 0.88 | 29.37 | ND | 35.40 | ND | 29.92 | 9.05 | 18.07 | 0.12 | 34.14 | ND | 31.75 | 0.85 |
| *Ltbp2* | 27.18 | 0.94 | 29.85 | 0.50 | 27.10 | 1.80 | 25.18 | 1.34 | 30.39 | 0.90 | 32.33 | 4.64 | 27.71 | 2.32 | 28.33 | 4.10 | 28.29 | 0.43 | 27.71 | 0.24 | 29.36 | 0.88 | 31.42 | 5.21 |
| *Mab21l2* | 34.60 | 0.28 | 34.70 | 0.41 | 32.38 | 0.27 | ND | ND | 35.52 | 0.53 | 36.04 | ND | 33.86 | 1.67 | 36.64 | ND | 33.59 | 3.27 | 32.61 | ND | 35.82 | ND | 38.66 | ND |
| *Mmp2* | 32.67 | 3.15 | 28.41 | 0.31 | 25.99 | 3.90 | 27.92 | ND | 29.16 | 0.46 | 24.77 | 5.38 | 39.53 | 0.06 | 28.12 | 0.78 | 29.79 | 5.02 | ND | ND | 29.31 | 0.47 | 33.97 | 8.38 |
| *Mstn* | 34.90 | 1.17 | ND | ND | 33.31 | 1.69 | 36.55 | 0.91 | ND | ND | 29.66 | 1.10 | 34.39 | 2.62 | 39.88 | ND | 31.92 | 1.80 | ND | ND | ND | ND | 30.23 | 2.99 |
| *Mthfr* | 35.93 | 2.59 | 32.78 | 5.01 | 33.39 | 5.87 | 33.30 | 4.11 | 33.32 | 4.67 | ND | ND | 33.31 | 2.06 | 28.70 | ND | 31.15 | 2.39 | 30.98 | 2.52 | ND | ND | 31.60 | 6.90 |
| *Nfatc1* | 38.26 | ND | ND | ND | 35.87 | ND | 38.88 | 1.84 | ND | ND | 25.28 | ND | 32.31 | 5.44 | 39.12 | ND | 31.61 | 2.38 | 27.88 | 3.42 | ND | ND | 28.04 | 0.20 |
| *Nog* | 28.65 | 1.49 | 32.98 | 1.11 | 25.88 | 6.12 | 27.58 | 2.56 | 33.71 | 1.53 | 29.64 | 1.78 | 26.00 | 6.40 | 26.09 | 7.23 | 22.40 | 9.14 | 20.77 | 1.88 | 32.39 | 3.80 | 31.94 | 0.47 |
| *Nos3* | 31.47 | 1.38 | 32.95 | 2.22 | 30.79 | 2.74 | 30.11 | 1.10 | 33.52 | 1.90 | 32.35 | 2.17 | 31.20 | 1.94 | 31.60 | 0.57 | 28.93 | 9.05 | 29.07 | 0.33 | 34.91 | 3.30 | 32.02 | ND |
| *Npy* | 34.78 | 5.62 | ND | ND | ND | ND | 32.34 | 4.67 | ND | ND | 37.49 | 0.79 | 31.14 | 12.27 | 34.52 | 3.52 | 27.71 | 8.34 | 31.41 | 1.07 | 36.51 | 3.20 | 31.51 | ND |
| *Nr3c1* | 32.99 | 1.60 | 35.99 | 0.96 | 30.44 | 7.46 | 32.55 | 0.86 | 36.60 | 1.39 | 34.73 | ND | 32.65 | 3.96 | 37.84 | ND | 20.92 | ND | 18.84 | 0.12 | 37.30 | 3.06 | 31.23 | 2.05 |
| *P2rx7* | 34.17 | 3.37 | 32.33 | 4.42 | 30.56 | 3.03 | 33.08 | 2.35 | 32.94 | 4.80 | 29.77 | 1.71 | 32.65 | 1.97 | 29.57 | 0.69 | 31.10 | 4.43 | 32.21 | 3.29 | 34.53 | 1.59 | 28.64 | 2.61 |
| *P3h1* | 31.08 | 1.09 | 36.42 | 3.55 | 33.22 | 0.75 | 28.09 | 2.76 | 35.72 | 4.58 | 35.34 | 1.80 | 31.07 | 3.72 | 36.30 | ND | 31.34 | 8.65 | 26.23 | 7.35 | 35.91 | 2.95 | 31.71 | 6.39 |
| *Plod2* | 33.48 | 6.47 | ND | ND | 33.03 | 2.51 | 35.82 | 3.57 | ND | ND | 30.70 | 2.36 | 38.34 | ND | ND | ND | 33.89 | ND | ND | ND | ND | ND | 30.44 | 0.94 |
| *Prl* | 35.83 | 4.67 | 37.81 | ND | 37.47 | ND | 35.90 | 5.46 | 37.96 | ND | 32.63 | 3.48 | 39.38 | ND | 39.50 | ND | 19.38 | 1.98 | 29.17 | 0.68 | 33.19 | ND | 35.54 | ND |
| *Pth* | 30.70 | 1.71 | 36.39 | 0.22 | 33.91 | 0.99 | 30.73 | 1.09 | 37.02 | 0.58 | 32.37 | 4.07 | 31.43 | 2.55 | 30.76 | ND | 31.29 | 2.39 | 28.21 | 3.82 | 34.32 | 0.59 | 32.13 | 6.25 |
| *Pth1r* | 33.35 | 1.89 | 33.08 | 1.87 | 31.04 | 3.45 | 33.71 | 0.42 | 33.77 | 1.46 | 32.96 | 0.36 | 31.47 | 5.65 | 35.36 | ND | 29.56 | 2.56 | 27.94 | 9.79 | 32.63 | 3.48 | 34.61 | ND |
| *Pthlh* | 31.97 | 1.01 | 37.61 | 0.44 | 35.31 | 1.74 | 29.30 | 4.19 | 38.36 | 0.75 | 31.93 | 2.92 | 31.90 | 1.64 | 37.35 | 0.75 | 33.04 | 6.12 | 25.96 | 2.30 | 34.49 | 0.88 | 32.54 | 1.49 |
| *Runx2* | 32.94 | 5.07 | 30.82 | 2.08 | 32.82 | 5.12 | 33.71 | 3.84 | 31.41 | 2.49 | 26.24 | 4.35 | 38.39 | ND | 29.77 | 1.72 | 30.02 | 1.46 | 26.73 | 5.77 | 35.00 | ND | 24.91 | 4.31 |
| *Sfrp1* | 36.23 | 1.32 | 35.83 | 0.48 | 33.43 | 4.26 | 31.98 | 6.00 | 36.89 | 0.64 | 32.00 | 2.76 | 35.36 | 3.01 | 38.29 | 0.20 | 31.82 | 9.44 | 29.33 | 0.09 | 32.39 | ND | 29.62 | 0.97 |
| *Sfrp4* | 33.70 | 2.07 | ND | ND | 32.91 | 4.67 | 34.75 | 2.08 | ND | ND | 23.96 | 0.08 | 31.82 | 5.67 | 36.29 | 0.20 | 32.80 | 2.77 | 34.47 | ND | 31.22 | 1.63 | 35.87 | 6.93 |
| *Shbg* | 34.78 | 2.33 | 38.87 | 0.76 | 34.17 | 2.31 | 23.41 | 8.76 | 39.08 | 1.12 | 34.96 | 1.22 | 31.74 | 5.68 | ND | ND | 28.16 | 6.67 | 23.17 | 8.99 | 39.40 | ND | 24.26 | 0.58 |
| *Sost* | 31.57 | 1.47 | 36.61 | 3.43 | 31.91 | 3.00 | 30.75 | 1.77 | 36.04 | 4.48 | 26.97 | 1.90 | 29.43 | 0.24 | 31.14 | 0.24 | 26.61 | 0.48 | 24.92 | 12.81 | 35.02 | 3.74 | 25.53 | 2.86 |
| *Sparc* | 33.94 | 0.57 | 29.02 | 5.14 | 26.53 | 4.73 | 30.09 | 5.11 | 29.67 | 4.75 | 24.72 | ND | 34.06 | 5.48 | 30.09 | 6.02 | 25.92 | 6.70 | 25.68 | 2.72 | 28.93 | 2.46 | 30.90 | ND |
| *Spp1* | 34.00 | 2.90 | 31.63 | 4.33 | 33.56 | 4.83 | 35.03 | 2.95 | 32.29 | 3.94 | 39.29 | 0.32 | 32.87 | 2.27 | 35.59 | 1.42 | 26.25 | 5.78 | 23.47 | ND | 34.69 | 6.34 | 37.18 | 1.69 |
| *Stat1* | 34.90 | ND | 32.46 | 0.56 | 24.04 | ND | 27.63 | 2.56 | 33.09 | 0.39 | 31.07 | 2.39 | 34.38 | 1.74 | 25.33 | 1.00 | 31.31 | 4.72 | 24.72 | 0.24 | 29.51 | ND | 31.08 | 2.73 |
| *Tgfb1* | ND | ND | 27.26 | 0.44 | 31.54 | 6.98 | 25.67 | 0.71 | 27.88 | 0.40 | 30.97 | 6.20 | 28.22 | 0.18 | 29.41 | 4.10 | 32.91 | 4.75 | 36.10 | 1.50 | 29.20 | 2.23 | 28.28 | 1.58 |
| *Timp2* | 34.67 | 1.47 | 29.00 | 0.63 | 29.84 | 6.27 | 31.92 | 6.57 | 29.57 | 0.45 | 30.19 | ND | 33.35 | 3.29 | 31.60 | 0.46 | 30.47 | 10.66 | 24.39 | 0.42 | 30.04 | ND | 33.52 | 1.81 |
| *Tnfaip3* | ND | ND | 28.62 | 0.35 | 24.60 | ND | ND | ND | 29.48 | 0.54 | 31.40 | 2.48 | ND | ND | 28.55 | 0.54 | 37.84 | 1.72 | ND | ND | 30.24 | ND | 35.14 | 3.93 |
| *Tnfrsf11a* | 34.85 | 1.44 | 35.88 | 0.93 | 37.36 | 3.20 | 32.69 | 1.12 | 36.58 | 1.41 | 25.31 | ND | 33.09 | 2.85 | 37.79 | 2.05 | 32.21 | 1.64 | 30.63 | 1.31 | 36.22 | ND | 27.56 | 2.98 |
| *Tnfrsf11b* | 32.58 | 2.47 | 33.56 | 2.28 | 31.20 | 2.60 | 32.07 | 5.48 | 34.12 | 1.96 | 31.71 | 3.01 | 33.25 | 1.67 | 36.75 | 1.36 | 29.26 | 0.03 | 34.49 | 6.61 | 35.48 | 3.46 | 33.26 | 1.73 |
| *Tnfrsf1b* | 34.61 | 2.61 | 29.46 | 0.30 | 28.08 | 0.96 | 33.13 | 1.69 | 30.28 | 0.52 | 24.90 | 0.01 | 29.89 | 2.42 | ND | ND | 33.88 | 2.18 | 28.88 | 5.13 | 37.63 | 1.99 | 22.13 | 0.02 |
| *Tnfsf11* | 30.19 | 1.32 | 35.43 | 0.29 | 32.08 | 2.84 | 30.01 | 2.02 | 36.10 | 0.55 | 30.79 | 2.50 | 27.26 | 0.41 | 34.66 | ND | 31.11 | 1.98 | 24.67 | 0.98 | 33.65 | 0.81 | 27.15 | ND |
| *Tshr* | 31.49 | 1.23 | 36.73 | 1.25 | 32.10 | 3.29 | 31.02 | 8.01 | 37.46 | 1.77 | 34.27 | 0.51 | 31.14 | 4.31 | 34.97 | ND | 31.25 | 4.26 | 34.86 | 6.81 | 37.51 | 1.13 | 38.33 | 0.51 |
| *Twist1* | 32.51 | 2.34 | 33.78 | 2.01 | 29.77 | 6.47 | 28.37 | 0.25 | 34.36 | 1.67 | 23.84 | 5.74 | 34.22 | 1.79 | 33.96 | 0.88 | 25.40 | 12.24 | 28.40 | ND | 35.22 | 3.44 | 25.51 | 7.37 |
| *Vdr* | 30.80 | 1.77 | 33.90 | 1.14 | 31.74 | 3.15 | 31.81 | 5.06 | 34.47 | 0.84 | 22.25 | 1.37 | 31.99 | 2.23 | 32.72 | ND | 29.34 | 8.63 | 33.95 | 6.26 | 33.45 | 1.58 | 24.68 | 4.87 |
| *Vegfa* | 25.15 | 2.28 | 26.63 | 0.31 | 24.59 | 0.34 | 25.09 | 0.35 | 27.27 | 0.47 | 24.09 | 0.66 | 26.39 | 1.17 | 24.82 | 0.33 | 19.11 | 0.11 | 21.24 | 1.51 | 26.90 | 1.24 | 24.16 | 0.79 |
| *Wnt10b* | 34.61 | 3.14 | 36.44 | ND | 32.56 | 4.69 | 28.33 | 5.46 | 36.60 | ND | 27.37 | 1.63 | 35.72 | 0.49 | ND | ND | 32.53 | 8.39 | 19.69 | ND | 33.01 | ND | 28.74 | 2.31 |
| *Wnt3a* | 35.27 | 0.92 | 35.47 | 2.23 | 34.49 | 0.92 | 30.59 | 9.65 | 36.27 | 1.75 | 20.86 | 1.10 | 32.62 | 6.15 | 38.63 | 0.71 | 19.09 | 0.02 | 19.78 | 0.46 | 36.11 | 4.97 | 28.25 | 9.95 |

*Weeks from the start of the experiment. ND = Non-Detectable. Ct values are the means of 3 animals for each time and groups. The same animals were sampled at 5, 7 and 10 weeks.

**Supplementary Table 4 ­– Quantification of cytokines in circulating EVs from control, OVX and HL-TS mice** using the Mouse XL Cytokine Array Kit (#ARY028, R&D Systems).

| **Protein** | **Intact mice** | | | **Sham-operated mice** | | | **OVX mice** | | | **HL-TS mice** | | |
| --- | --- | --- | --- | --- | --- | --- | --- | --- | --- | --- | --- | --- |
|  | **Weeks** | | | **Weeks** | | | **Weeks** | | | **Weeks** | | |
|  | 5 | 7 | 10 | 5 | 7 | 10 | 5 | 7 | 10 | 5 | 7 | 10 |
|  | **Pixel^*^** | **Pixel** | **Pixel** | **Pixel** | **Pixel** | **Pixel** | **Pixel** | **Pixel** | **Pixel** | **Pixel** | **Pixel** | **Pixel** |
| Adiponectin | ND | ND | 12734 | ND | ND | 12900 | ND | ND | 24890 | 4696 | 4813 | 26674 |
| Amphiregulin | 5749 | 6418 | 35140 | 6066 | 6206 | 35597 | 8216 | 9513 | 46491 | 7319 | 8705 | 49469 |
| Angiopoieitin 1 | 5362 | 5496 | 24576 | 12054 | 12054 | 24895 | 2485 | 3742 | 58131 | 5476 | 7431 | 47334 |
| Angiopoieitin 2 | 5906 | 6054 | 23153 | 6862 | 8427 | 23454 | 8144 | 9491 | 43760 | 7916 | 9296 | 34053 |
| Angiopoieitin-like 3 | 5825 | 5971 | 25719 | 9119 | 10357 | 26053 | 11288 | 12644 | 53996 | 6768 | 8486 | 37693 |
| C5A | ND | ND | 13969 | ND | ND | 14151 | ND | ND | 41792 | ND | ND | 43332 |
| CCL 2 | ND | ND | 15002 | ND | ND | 15197 | ND | ND | 32686 | ND | ND | 36059 |
| CCL 3 | 5542 | 5681 | 32706 | 10268 | 11554 | 30599 | 432 | 2046 | 24874 | 8780 | 10246 | 26072 |
| CCL 6 | ND | ND | 15726 | ND | ND | 15930 | ND | ND | 45887 | ND | ND | 44421 |
| CCL 11 | 203 | 1136 | 33238 | 259 | 1965 | 36061 | 18235 | 20694 | 54460 | 10918 | 12667 | 49942 |
| CCL 12 | 8761 | 10251 | 30964 | 6227 | 6527 | 31367 | 9982 | 11541 | 54652 | 6769 | 8085 | 57293 |
| CCL 17 | 7898 | 9418 | 39091 | 8147 | 9660 | 39599 | 10310 | 11867 | 70300 | 8291 | 9404 | 48626 |
| CCL 19 | 9338 | 12163 | 33429 | 2338 | 5028 | 33863 | 12338 | 14894 | 59299 | 11584 | 14811 | 56724 |
| CCL 21 | ND | ND | 19309 | ND | ND | 19560 | ND | ND | ND | ND | ND | 26271 |
| CCL 22 | 7073 | 7249 | 38554 | 10848 | 11991 | 39055 | 7586 | 9626 | 53407 | 9587 | 11437 | 61478 |
| CD 14 | 3515 | 3603 | 24345 | 6142 | 7362 | 24661 | 4087 | 5354 | 50167 | 5269 | 5401 | 48762 |
| CD 26 | ND | ND | 25771 | ND | ND | 26106 | ND | ND | 42291 | ND | ND | 38684 |
| CD 40 | 4710 | 4827 | 24589 | 10741 | 12332 | 24908 | 13849 | 15434 | 47352 | 8479 | 8691 | 40178 |
| CD 93 | ND | ND | 21872 | ND | ND | 22156 | ND | ND | 32095 | ND | ND | 27075 |
| CD 142 | ND | 1425 | 29818 | ND | 1159 | 30205 | 5316 | 6636 | 49927 | 25435 | 28254 | 38040 |
| CD 160 | 2720 | 2787 | 34756 | 2460 | 2910 | 35207 | 2657 | 2667 | 31328 | 10137 | 11677 | 50076 |
| Chemerin | 7847 | 11947 | 48934 | 4578 | 6321 | 49570 | 12498 | 14458 | 77310 | 31710 | 36905 | 64695 |
| Chitinase 3-like1 | 5457 | 5593 | 19447 | 12009 | 11309 | 19700 | 11386 | 11386 | 39250 | 6026 | 6001 | 42111 |
| Complement factor D | ND | ND | 22176 | ND | ND | 22464 | ND | 753 | 36596 | ND | 1613 | 39617 |
| CX3CL1 | ND | 700 | 27705 | ND | 884 | 28065 | ND | 1123 | 46719 | ND | 1393 | 40495 |
| CXCL1 | ND | ND | ND | ND | ND | ND | ND | ND | ND | ND | ND | 39078 |
| CXCL2 | 4727 | 5647 | 41675 | 5221 | 7140 | 42216 | 5142 | 7014 | 43629 | 5465 | 6827 | 56989 |
| CXCL9 | ND | 1792 | 31955 | ND | 1321 | 32370 | 0 | 1228 | 63601 | 3372 | 1872 | 75582 |
| CXCL10 | ND | ND | 11928 | ND | ND | 12083 | ND | ND | 42677 | ND | ND | 53616 |
| CXCL11 | ND | ND | 33262 | ND | ND | 33694 | ND | ND | 63332 | 5290 | 6493 | 61604 |
| CXCL13 | ND | ND | 19056 | ND | ND | 19303 | 0 | 818 | 52313 | 0 | 0 | 47407 |
| CXCL16 | 0 | 0 | 24705 | 0 | 0 | 26242 | 2535 | 2535 | 56094 | 4518 | 6020 | 41586 |
| Cystatin C | 4101 | 4203 | 32292 | 3626 | 3626 | 37777 | 4013 | 4818 | 56449 | 5827 | 6579 | 54822 |
| DKK-1 | 1978 | 1690 | 55519 | 4913 | 4903 | 35981 | 4332 | 4392 | 45166 | 1997 | 1648 | 51978 |
| DPP4 | ND | ND | ND | ND | ND | ND | ND | ND | ND | ND | 894 | ND |
| EGF | ND | ND | 11679 | ND | ND | 11830 | ND | ND | 42123 | ND | 1022 | 58521 |
| Endoglin | ND | ND | ND | ND | ND | ND | ND | ND | 31049 | ND | 816 | 52373 |
| Endostatin | 3671 | 4750 | 38129 | 5461 | 6909 | 38625 | 9588 | 10298 | 62813 | 6033 | 12502 | 70620 |
| Fetuin A | 1804 | 2856 | 66113 | 1764 | 2392 | 70011 | 8276 | 9512 | 73716 | 5671 | 7101 | 64750 |
| FGF-1 | ND | ND | 19488 | ND | ND | 19741 | ND | ND | 42572 | 4888 | 6389 | 45132 |
| FGF-21 | ND | ND | 36785 | ND | ND | 37263 | ND | ND | 54176 | 6361 | 7605 | 62146 |
| FLT3 Ligand | ND | ND | 23441 | ND | ND | 23745 | 11083 | 14083 | 51629 | 5343 | 5806 | 45341 |
| GAS 6 | ND | ND | 27838 | ND | ND | 28200 | 4772 | 5772 | 49374 | 6541 | 7127 | 51519 |
| G-CSF | 17950 | 17622 | ND | 17607 | 20602 | ND | 17332 | 18904 | 68685 | 20094 | 14992 | 101743 |
| GDF-15 | ND | 522 | 18032 | ND | 655 | 18266 | 8239 | 824 | 43578 | ND | 691 | 44139 |
| GM-CSF | ND | ND | ND | ND | ND | ND | ND | ND | 39127 | ND | 901 | 51627 |
| HGF | ND | ND | 20424 | ND | ND | 20689 | ND | ND | 43375 | ND | ND | 46559 |
| ICAM-1 | ND | ND | 15594 | ND | ND | 14774 | ND | ND | 32366 | ND | ND | 31783 |
| IFN-γ | ND | ND | 11853 | ND | ND | 12007 | 3381 | 3381 | 47805 | ND | 895 | 57503 |
| IGFBP-2 | ND | 1043 | 50540 | ND | 967 | 51197 | 2700 | 2700 | 58770 | 0 | 990 | 45792 |
| IGFBP-3 | ND | ND | ND | ND | ND | ND | ND | ND | 31528 | ND | ND | 28065 |
| IGFBP-5 | ND | ND | 24093 | ND | ND | 24406 | ND | ND | 46016 | ND | 1312 | 40876 |
| IGFBP-6 | ND | 1151 | 29061 | ND | 1345 | 29439 | 6224 | 7075 | 58222 | ND | 8151 | 65382 |
| IL-1α | 4012 | 4089 | 59346 | 9089 | 10649 | 60117 | 16184 | 17550 | 79613 | 8088 | 9713 | 83079 |
| IL-1β | ND | ND | 987 | ND | ND | 799 | ND | ND | 34582 | ND | ND | 44517 |
| IL-1 Receptor antagonist | 189- | 1357 | 38436 | 9295 | 10819 | 34884 | 16014 | 19972 | 78199 | 4039 | 6232 | 101743 |
| IL-3 | ND | ND | 10484 | ND | ND | 10620 | ND | ND | 30709 | ND | ND | 42329 |
| IL-4 | ND | ND | 36117 | ND | ND | 36586 | ND | ND | 44070 | ND | 1214 | 49806 |
| IL-5 | ND | ND | 27052 | ND | ND | 27404 | ND | ND | 42673 | ND | 943 | 57290 |
| IL-6 | ND | ND | ND | ND | ND | ND | ND | ND | 2930 | ND | ND | 3631 |
| IL-7 | ND | 1910 | 65135 | ND | 2356 | 65981 | 6733 | 8109 | 87664 | ND | 1847 | 76038 |
| IL-10 | 8476 | 10241 | 50625 | 15574 | 15574 | 51283 | 10092 | 11956 | 62324 | 11232 | 12857 | 2345 |
| IL-11 | 8965 | 11011 | 62847 | 5259 | 6778 | 53534 | 5856 | 8038 | 49528 | 11134 | 14020 | 65738 |
| IL-12 | 5812 | 5957 | 34117 | 8166 | 9534 | 34560 | 8082 | 8082 | 51852 | 7489 | 8875 | 54396 |
| IL-13 | ND | 3989 | 23451 | ND | 1104 | 23755 | 6297 | 6297 | 61194 | 3972 | 4071 | 55368 |
| IL-15 | 12073 | 12374 | 44634 | 16438 | 19751 | 45214 | 4784 | 6073 | 74681 | 20432 | 22452 | 72279 |
| IL-22 | ND | ND | 19871 | ND | ND | 20129 | ND | ND | 40076 | ND | ND | 41121 |
| IL-23 | 5849 | 4970 | 27608 | 4889 | 4989 | 27966 | 4389 | 4289 | 21894 | 5089 | 5308 | 28279 |
| IL-27 | ND | 919 | 35850 | ND | 1088 | 36316 | ND | 846 | 36804 | ND | 863 | 53281 |
| IL-28A | 6154 | 7509 | 25977 | 9376 | 10822 | 26314 | 9104 | 10969 | 58606 | 9930 | 11483 | 110075 |
| IL-33 | 21732 | 24495 | 45840 | 22657 | 26139 | 46435 | 5014 | 7125 | 76443 | 22818 | 25305 | 80577 |
| LDL R | ND | 1475 | 57427 | ND | 1127 | 58174 | ND | 1309 | 62787 | ND | 756 | 55709 |
| Leptin | 5434 | 6106 | 33582 | 7847 | 9278 | 34322 | 10155 | 11617 | 52595 | 7709 | 7901 | ND |
| LIF | 16855 | 19479 | 68596 | 16569 | 18904 | 69487 | 17514 | 20551 | 90698 | 22974 | 25227 | 68934 |
| Lipocalin 2 | ND | ND | ND | ND | ND | ND | ND | ND | ND | ND | 1136 | 31632 |
| LIX | 10968 | 12065 | 19411 | 9831 | 11616 | 19663 | 13872 | 14665 | 51907 | 6903 | 8179 | 49801 |
| M-CSF | 4985 | 5909 | 41633 | 10483 | 11914 | 42174 | 11500 | 12646 | 52582 | 5620 | 6673 | 56972 |
| MMP2 | 7332 | 8294 | 68296 | 9670 | 11456 | 69183 | 9704 | 11318 | 64431 | 7775 | 9687 | 92476 |
| MMP-3 | ND | ND | ND | ND | ND | ND | ND | ND | ND | ND | ND | 42154 |
| Myeloperoxidase | 4159 | 5352 | 51056 | 8714 | 10597 | 51719 | 6867 | 9510 | 68621 | 7262 | 9465 | 68270 |
| Osteoprotegerin | ND | ND | 12436 | ND | ND | 11653 | ND | ND | 564 | ND | ND | 32767 |
| Osteopontin | ND | ND | 24289 | ND | ND | 25618 | ND | ND | ND | ND | ND | 39693 |
| PD-ECGF | ND | ND | 33108 | ND | ND | 33538 | ND | ND | 44313 | ND | ND | 52079 |
| PDGF-BB | ND | ND | 29521 | ND | ND | 29905 | ND | ND | 44009 | 19016 | 19491 | 39984 |
| Pentraxin 2 | ND | ND | 38982 | ND | ND | 39488 | ND | ND | 41687 | 6861 | 7033 | 34680 |
| Pentratrix 3 | 8710 | 9844 | 40015 | 5906 | 7669 | 40535 | 10474 | 11823 | 77561 | 15856 | 18830 | 87877 |
| Periostin | ND | ND | ND | ND | ND | ND | ND | ND | 36917 | ND | ND | 29587 |
| PREF-1 | 6691 | 8146 | 41256 | 11284 | 11284 | 41792 | 9540 | 9540 | 59714 | 7261 | 9603 | 72805 |
| Proliferin | 6366 | 7520 | 24562 | 7702 | 7702 | 24881 | 12568 | 12568 | 49815 | 9812 | 12520 | 60718 |
| Proprotein convertase 9 | 6713 | 8421 | 27370 | 7544 | 9217 | 27725 | 10177 | 11905 | 54605 | 14819 | 17294 | 59411 |
| P-Selectin | ND | ND | ND | ND | ND | ND | ND | ND | 34792 | ND | ND | ND |
| RAGE | 6155 | 6309 | 24075 | 4845 | 6084 | 24387 | 9674 | 11262 | 43917 | 7286 | 8855 | 46870 |
| RBP4 | ND | 377 | 14603 | ND | 794 | 14792 | ND | 728 | 38320 | ND | 1304 | 32256 |
| REG3G | ND | ND | 29493 | ND | ND | 29876 | ND | 1123 | 45718 | 4684 | 4801 | 37128 |
| Resistin | ND | ND | 12203 | ND | ND | 12361 | ND | ND | 29904 | ND | ND | 34581 |
| Serpin E1 | 3666 | 3758 | 46101 | 6861 | 6861 | 46700 | 6958 | 6958 | 58274 | 15702 | 17706 | 38226 |
| Serpin F1 | 3296 | 3378 | 22833 | 4522 | 4522 | 23129 | 4183 | 4183 | 45132 | 5739 | 7546 | 39552 |
| TIM-1 | 7447 | 8943 | 29072 | 5449 | 5449 | 29450 | 12163 | 13278 | 44211 | 11222 | 11503 | 48368 |
| TNF-α | ND | ND | 25091 | ND | ND | 21365 | ND | ND | ND | ND | ND | 35799 |
| TNSF123B | ND | ND | 11879 | ND | ND | 12033 | ND | ND | 28198 | ND | ND | 32753 |
| Thrombopoietin | ND | ND | 25971 | ND | ND | 26308 | ND | ND | 33750 | ND | 1362 | 43100 |
| VCAM-1 | ND | ND | 23260 | ND | ND | 23562 | ND | ND | 52409 | 4375 | 4484 | 34093 |
| VEGF | 5208 | 5608 | 44552 | 5219 | 5619 | 46346 | 6671 | 6738 | 40209 | 8671 | 8971 | 35617 |
| WISP-1 | ND | ND | 31604 | ND | ND | 32015 | ND | ND | 43404 | ND | ND | 49293 |

* = Values are pixel intensity of a pool of 3 different samples, no SD available. ND = Non-Detectable. The same animals ware sampled at 5, 7 and 10 weeks.

**
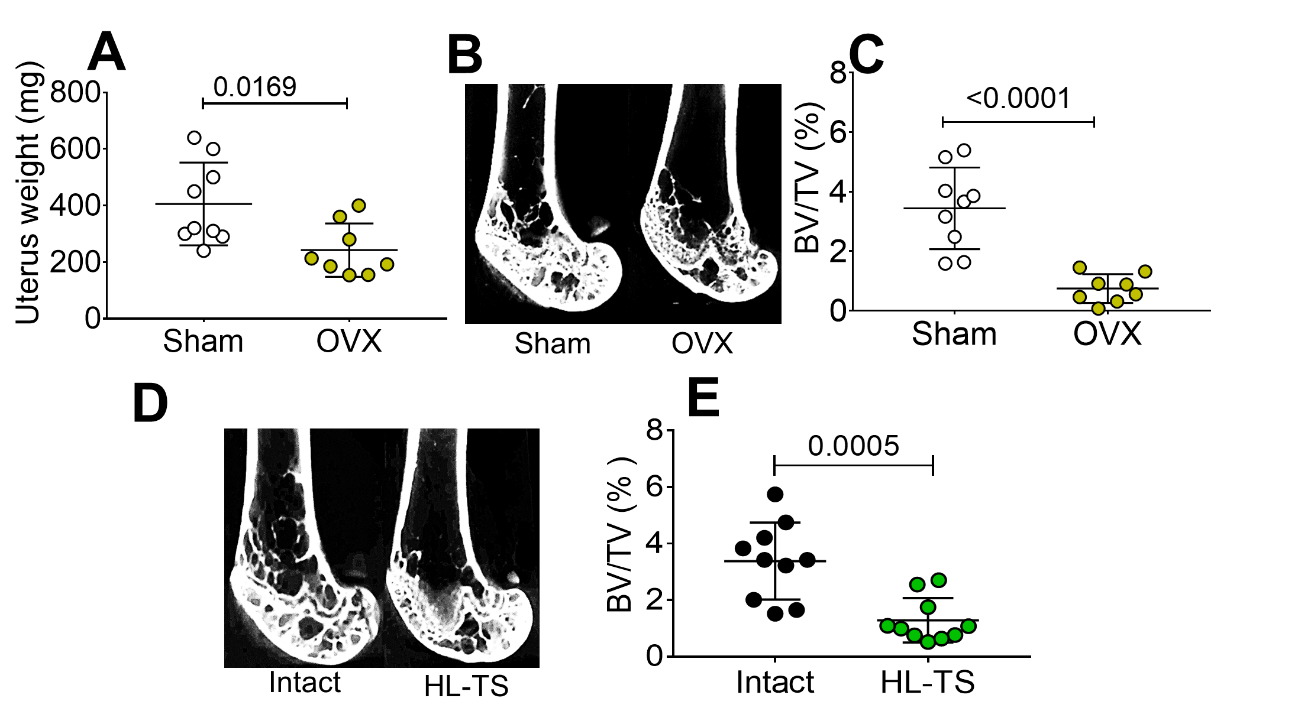
**

**Figure S1: Induction of osteoporotic phenotype in OVX and HL-TS models.** **(A)** Weights of the uteri of Sham and OVX mice at the end of the experiment. **(B)** Representative μCT images and **(C)** quantification of bone volume/tissue volume (BV/TV) of femurs from Sham and ovariectomized (OVX) mice at the end of experiments. **(D)** Representative μCT images and **(E)** quantification of bone volume/tissue volume (BV/TV) of femurs from Intact and hind limb-tail suspended (HL-TS) mice at the end of experiment. Results are the mean±SD. Statistics: unpaired Student’s t-test. p values are reported in the graphs.

**
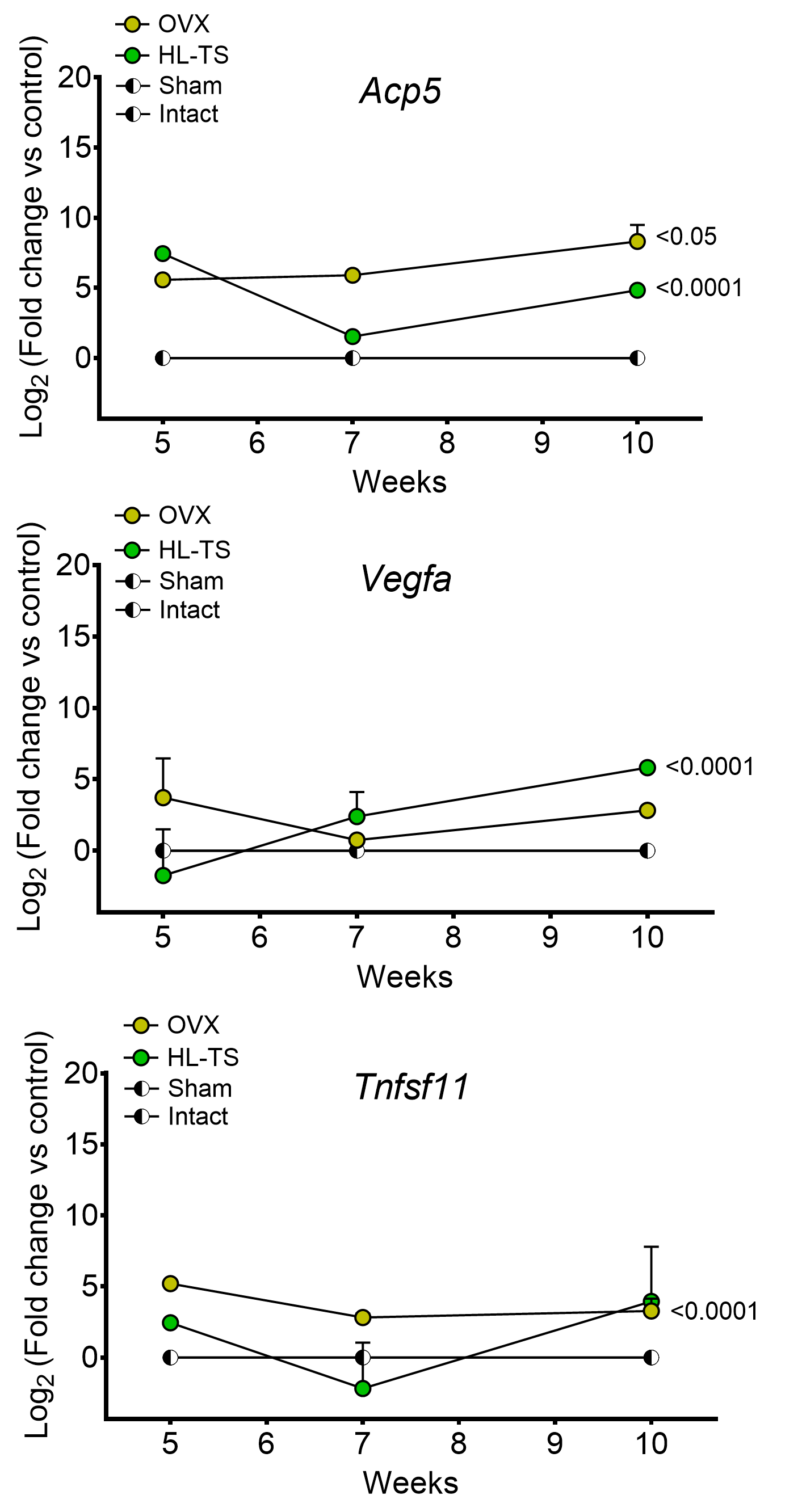
**

**Figure S2: Evaluation of shared transcripts in circulating EVs.** Time-dependent expression of shared genes in circulating EVs from OVX vs Sham and HL-TS vs Intact. Data are the mean + SD of 3 animals per group. Statistics: one-way ANOVA using non-parametric Dunnet post-hoc test among multiple groups (whole curves OVX vs Sham and HL-TS vs Intact mice). p values are shown in the graphs


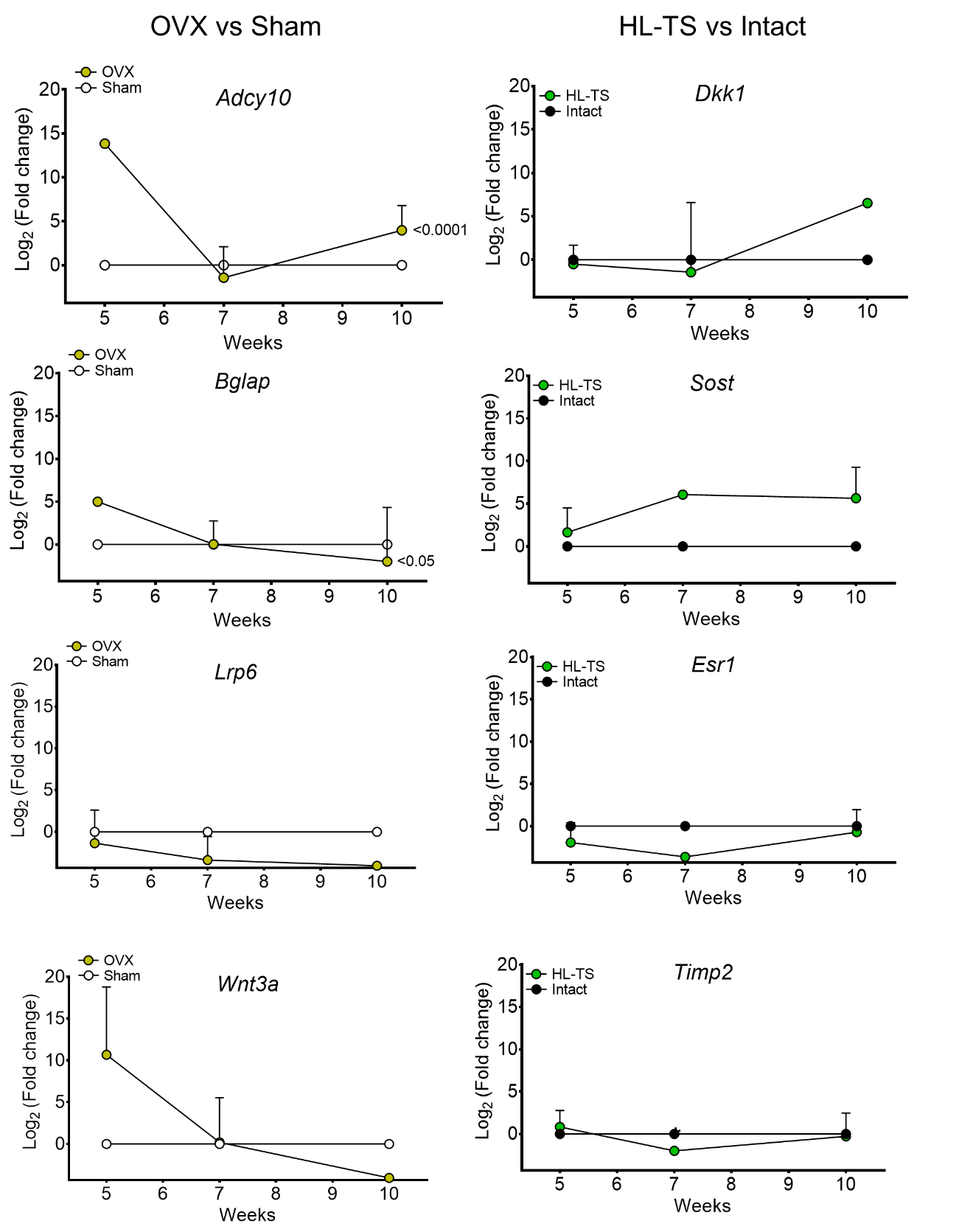


**Figure S3: Evaluation of specific transcripts in circulating EVs.** Time-dependent expression of genes specifically identified in circulating EVs from OVX vs Sham and HL-TS vs Intact. Data are the mean + SD of 3 animals per group. Statistics: one-way ANOVA using non-parametric Dunnet post-hoc test among multiple groups (whole curves OVX vs Sham and HL-TS vs Intact mice). p values are shown in the graphs

**
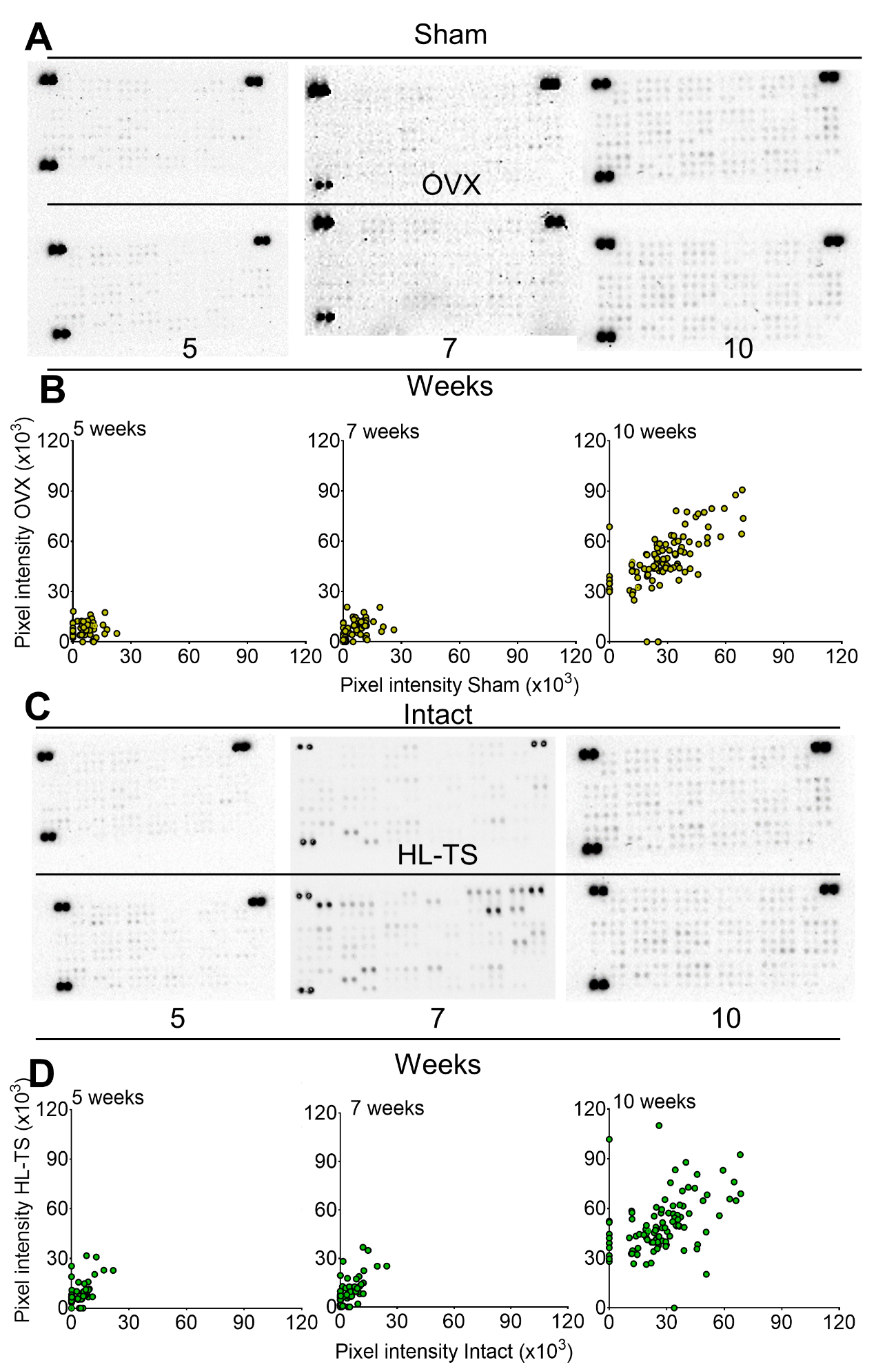
**

**Figure S4: Evaluation of cytokine content in circulating EVs.** **(A)** Images showing membranes of the Cytokines Array incubated with pools of n=3 samples of proteins extracted from circulating EVs of Sham and OVX mice at the indicated time. **(B)** Quantification of pixel intensity (Arbitrary Units) of the membranes shown in (A). **(C)** Images showing membranes of the Cytokines Array incubated with pools of n=3 samples of proteins extracted from circulating EVs of Intact and HL-TS mice at indicated time. **(D)** Quantification of pixel intensity (Arbitrary Units) of the membranes shown in **(C)**.
